# Crosstalk between the serine/threonine kinase StkP and the response regulator ComE controls the stress response and intracellular survival of *Streptococcus pneumoniae*

**DOI:** 10.1371/journal.ppat.1007118

**Published:** 2018-06-08

**Authors:** Germán E. Piñas, Nicolás M. Reinoso-Vizcaino, Nubia Y. Yandar Barahona, Paulo R. Cortes, Rosario Duran, Chandan Badapanda, Ankita Rathore, Dario R. Bichara, Melina B. Cian, Nadia B. Olivero, Daniel R. Perez, José Echenique

**Affiliations:** 1 Departamento de Bioquímica Clínica—CIBICI (CONICET), Facultad de Ciencias Químicas, Universidad Nacional de Córdoba, Córdoba, Argentina; 2 Department of Biology, University of Utah, Salt Lake City, Utah, United States of America; 3 Instituto Pasteur de Montevideo and Instituto de Investigaciones Biológicas Clemente Estable, Unidad de Bioquímica y Proteómica Analíticas, Montevideo, Uruguay; 4 Bioinformatics Division, Xcelris Lab Limited, Ahmedabad, India; 5 Fundacion Instituto Leloir, CONICET, Buenos Aires, Argentina; 6 Department of Population Health, College of Veterinary Medicine, University of Georgia, Athens, Georgia, United States of America; University of Birmingham, UNITED KINGDOM

## Abstract

*Streptococcus pneumoniae* is an opportunistic human bacterial pathogen that usually colonizes the upper respiratory tract, but the invasion and survival mechanism in respiratory epithelial cells remains elusive. Previously, we described that acidic stress-induced lysis (ASIL) and intracellular survival are controlled by ComE through a yet unknown activation mechanism under acidic conditions, which is independent of the ComD histidine kinase that activates this response regulator for competence development at pH 7.8. Here, we demonstrate that the serine/threonine kinase StkP is essential for ASIL, and show that StkP phosphorylates ComE at Thr^128^. Molecular dynamic simulations predicted that Thr^128^-phosphorylation induces conformational changes on ComE’s DNA-binding domain. Using nonphosphorylatable (ComE^T128A^) and phosphomimetic (ComE^T128E^) proteins, we confirmed that Thr^128^-phosphorylation increased the DNA-binding affinity of ComE. The non-phosphorylated form of ComE interacted more strongly with StkP than the phosphomimetic form at acidic pH, suggesting that pH facilitated crosstalk. To identify the ComE-regulated genes under acidic conditions, a comparative transcriptomic analysis was performed between the *comE*^*T128A*^ and *wt* strains, and differential expression of 104 genes involved in different cellular processes was detected, suggesting that the StkP/ComE pathway induced global changes in response to acidic stress. In the *comE*^*T128A*^ mutant, the repression of *spxB* and *sodA* correlated with decreased H_2_O_2_ production, whereas the reduced expression of *murN* correlated with an increased resistance to cell wall antibiotic-induced lysis, compatible with cell wall alterations. In the *comE*^*T128A*^ mutant, ASIL was blocked and acid tolerance response was higher compared to the *wt* strain. These phenotypes, accompanied with low H_2_O_2_ production_,_ are likely responsible for the increased survival in pneumocytes of the *comE*^*T128A*^ mutant. We propose that the StkP/ComE pathway controls the stress response, thus affecting the intracellular survival of *S*. *pneumoniae* in pneumocytes, one of the first barriers that this pathogen must cross to establish an infection.

## Author summary

*Streptococcus pneumoniae* is a major human pathogen and is the causal agent of otitis (media) and sinusitis. It is also responsible for severe infections such as bacteremia, pneumonia, and meningitis, associated with 2 million annual deaths. Although this bacterium is part of the human nasopharynx commensal microbiota, it can become a pathogen and cross the epithelial cell barrier to establishing infections of varying intensity. Although *S*. *pneumoniae* is considered to be a typical extracellular pathogen, transient intracellular life forms have been found in eukaryotic cells, suggesting a putative survival mechanism. Here, we report that the serine-threonine kinase StkP was able to phosphorylate the response regulator ComE to control different cellular processes in response to environmental stress. Moreover, the phosphorylation of ComE on Thr^128^, and the consequent conformational and functional changes resulting from this event, extended the current knowledge of molecular activation mechanisms of response regulators. In this report, we provide evidence for the regulatory control exerted by the StkP/ComE pathway on acid-induced autolysis (associated with pneumolysin release), the acid tolerance response, and H_2_O_2_ production to modulate tissue damage and intracellular survival, which are ultimately linked to pneumococcal pathogenesis.

## Introduction

Sensing and transducing external (or internal) signals into an appropriate physiological response is part of a microorganism strategy to survive in a constantly changing environment. Signal transduction is mainly carried out by protein kinases, which autophosphorylate upon sensing stimuli and then catalyze the phosphorylation of a specific substrate that initiates an adaptive cellular response. In prokaryotes, signaling pathways are mainly mediated by two-component systems (TCS) consisting of sensor histidine kinases (HK) that phosphorylate response regulators (RR) on a receiver domain, thereby activating the effector domains of these regulators to induce a physiological event in bacterial cells. Generally, the RR effector domains bind regions of DNA that control gene expression in response to environmental changes [1,2]. Each particular HK presents a remarkable specificity for its cognate RR and is capable of identifying particular RRs.

Eukaryotic-like Ser/Thr protein kinases (STKs) are also present in prokaryotes, where they play key roles in several cellular processes, including the central or secondary metabolism, developmental processes, cell division and virulence [3]. The major human bacterial pathogen *Streptococcus pneumoniae* (*S*. *pneumoniae* or the pneumococcus) encodes a single copy of StkP, a eukaryotic-like serine/threonine protein kinase gene [4]. StkP is a membrane protein composed of an N-terminal kinase domain facing the cytoplasm, a short transmembrane region, and an extracellular C-terminal region containing four PASTA (Penicillin-binding protein and Ser/Thr protein kinase Associated) domains [4–6]. Comparison of the global expression profile of the wild-type and Δ*stkP* strains has revealed that the transcription of genes involved in the cell wall metabolism, pyrimidine biosynthesis, DNA repair, iron uptake, and oxidative stress response are controlled by StkP, which explain why *stkP* mutations have pleiotropic effects [7]. It has also been described that StkP phosphorylates several target proteins, mainly on threonine residues, with PASTA domains being essential for kinase activity [8–10]. However, phosphorylation on serine residues seems to be independent of StkP [11]. Immunofluorescence microscopy of pneumococcal cells localized StkP to the cell division apparatus [12], with phenotypic studies having demonstrated its impact on several cellular functions [13,14]. In fact, the *stkP* mutant displayed morphological and growth defects, cell division alterations, increased LytA-dependent autolysis (induced by either antibiotics or growth at an alkaline pH of 7.8), reduced tolerance to stress conditions (including acidic stress), and pilus-mediated adherence in endothelial cells [4,7,11,15–17]. StkP is also essential for virulence, being necessary for lung infection and for invading and growing in the bloodstream of intranasally infected mice [4].

In *S*. *pneumoniae*, transient competence development (ability to take up exogenous DNA) in exponentially growing cells is considered a stress response to alkaline pH [2], with its core regulatory circuit being controlled by the TCS ComDE. In this quorum sensing system, the membrane-integrated HK ComD senses the extracellular accumulation of a 17 amino acid competence stimulating peptide (CSP)[2]. Upon activation by a critical concentration of CSP, ComD phosphorylates the response regulator ComE at Asp^58^ [18], which consequently initiates the transcription of *comCDE*, *comAB*, and *comX* (a gene encoding an alternative sigma factor) [19,20]. ComX turns on the transcription of genes whose products are involved in DNA binding, uptake, and recombination [21]. In this sense, the competence development is considered to be a type of stress response to alkaline pH [2]. It has been reported that StkP can also regulate competence at pH 7.8. Cells lacking StkP do not develop natural competence [4] and show severely reduced CSP-induced competence [7,22], despite having increased expression of many genes of the CSP-regulated competence regulon [7].

To invade tissues, *S*. *pneumoniae* must overcome a variety of stress situations, such as acidic pH, as a consequence of host inflammatory responses against the invading pathogen [23]. This characteristic local acidosis is caused by infiltration of neutrophils and activation of inflammatory cells, which leads to increased energy and oxygen demand, accelerated glucose consumption via glycolysis and thus increased lactic acid secretion [24–26]. For instance, pH values obtained from pleural fluids from patients with acute bacterial pneumonia caused by *S*. *pneumoniae* showed an acidic pH close to 6.80 [27]. Interestingly, the lowest pH value that *S*. *pneumoniae* has been shown to be tolerant to is around 4.4 in phagosomal vesicles during the first few minutes after phagocytosis [28]. Although *S*. *pneumoniae* is considered a typical extracellular pathogen, a transient intracellular life was described, suggesting that it can survive inside eukaryotic cells. *S*. *pneumoniae* can cross brain microvascular endothelial cells inside vesicles derived from early and/or late endosomes [29] [30]. It is well accepted that acidification is essential to endosome/lysosome maturation, with early endosomes having a pH in the 6.8–6.1 range, late endosomes in the 6.0–4.8 range, whereas lysosomal pH values can drop to 4.5 [31]. In the case of a putative endosomal survival, *S*. *pneumoniae* must survive acidic conditions. Martin-Galiano *et al* [32] described that *S*. *pneumoniae* is able to induce an acid tolerance response (ATR) mechanism. Previously, we also showed that F_0_.F_1_-ATPase, a proton pump that controls intracellular pH, is relevant for ATR induction in *S*. *pneumoniae*. In addition, we demonstrated that the F_0_.F_1_-ATPase and ATR are necessary for the intracellular survival of the pneumococcus in macrophages [33].

As part of the acidic stress response, we have reported that exposure of *S*. *pneumoniae* to acidic culture conditions triggers a lytic response by the major autolysin LytA. The acidic-stress induced lysis (ASIL) response is promoted by ComE and repressed by the CiaRH TCS. Despite requiring ComE, ASIL does not depend on CSP or ComD. Curiously, the *comE* gene is induced by acidic stress, but the competence-related ComX sigma factor, whose expression is regulated by ComE, does not participate in this signaling pathway [34]. We have also reported that ComDE and CiaRH control pneumococcal survival in pneumocytes in contrasting ways, CiaRH was essential for ATR and intracellular survival, whereas ComE repressed its activation. Moreover, ComE in a CSP-independent manner, was necessary for ASIL, whereas CiaRH protected against its induction by modulating LytA autolysin expression on the pneumococcal surface. These results suggest that both TCSs control the acidic stress response and establish either a survival or a suicidal response by independent pathways, either in acidified culture media or in pneumocyte cultures [33]. These findings indicate that ComE is activated under acidic conditions by an alternative signaling pathway that differs from the quorum sensing mechanism reported during competence development at alkaline pH. Alternatively, it was proposed that StkP is involved in competence at pH 7.8, by the fact that cells lacking StkP do not develop natural competence [4,7,22]. StkP is also essential for virulence, to establish infections in the lung and for invading and growing in the bloodstream of intranasally infected mice [4].

The main aim of this work was to elucidate whether ComE is part of a novel activation pathway used by *S*. *pneumoniae* to induce the acidic stress response and to control its intracellular survival mechanism in pneumocytes. Here, we demonstrate that StkP controls ComE activation by phosphorylation of the Thr^128^ residue of the latter, increasing both its dimerization capacity and its DNA-binding affinity. Under acidic conditions, the StkP/ComE HK-independent pathway regulated 104 genes involved in different cellular processes, such as H_2_O_2_ production and oxidative stress tolerance. The StkP/ComE pathway is independent of the HK-dependent ComD/ComE system, which regulates more than 180 genes at pH 7.8 [35]. The participation in HK-independent and HK-dependent stress regulatory systems places ComE as a global regulator. This newly discovered StkP/ComE signaling pathway triggered the acidic stress response by inducing ASIL and inhibiting ATR and the intracellular survival of *S*. *pneumoniae* in pneumocytes, one of the first barriers that this pathogen must overcome to establish an infection.

## Results

### The ComE response regulator is activated under acidic conditions by a histidine-kinase independent pathway

We previously reported that ComE was required in the acidic stress induced lysis (ASIL) mechanism, which was independent of its cognate histidine kinase ComD at pH 6.0 [34]. This is in contrast to the ComD/quorum-sensing dependence on ComE activation at pH 7.8 necessary for competence development [19]. This initial observation led us to investigate whether other pneumococcal TCS-associated HKs could be activating ComE by a crosstalk mechanism, as described for other bacteria [36]. We hypothesized that if other HKs were involved in ComE activation by phosphorylation, the corresponding *hk* mutant should display alterations in the ASIL induction. Thus, the lytic phenotype was determined under acidic stress conditions for all the pneumococcal *hk* mutants that we had previously constructed in the background of the R801 strain by insertion-duplication mutagenesis (S1 Table) [33]. We observed that all the *hk* mutants showed the same lytic response as the parental R801strain, indicating that none of the tested HKs was involved in ASIL (S2 Table). In addition, the *comD*^*F183X*^ mutant was used because it lacks the HK domain due to a *stop codon* at residue 163 (S2 Table) and therefore it is unable to activate ComE [33]. This mutant was constructed to avoid putative alterations in the *comCDE* operon expression, and it showed the same ASIL phenotype than the *ΔcomD* mutant (S2 Table), indicating that the truncated ComD protein expressed by the *comD*^*F183X*^ mutant has not impact on the ASIL activation. In order to avoid a putative residual effect of *comD*^*F183X*^ on the ComE activation, we determined ASIL in the double *comD*^*F183X*^ *hk* mutants, which were constructed by transforming the *comD*^*F183X*^ mutant with individual plasmids containing the different *hk* mutations. Despite the low transformability of the *comD*^*F183X*^ mutant, all the double *comD*^*F183X*^ *hk* mutants displayed the same ASIL phenotype as those obtained for each individual *hk* mutant (S2 Table). Taken together, these results indicated that HKs were not responsible for activation of ComE and the resulting ASIL.

**Fig 1:**
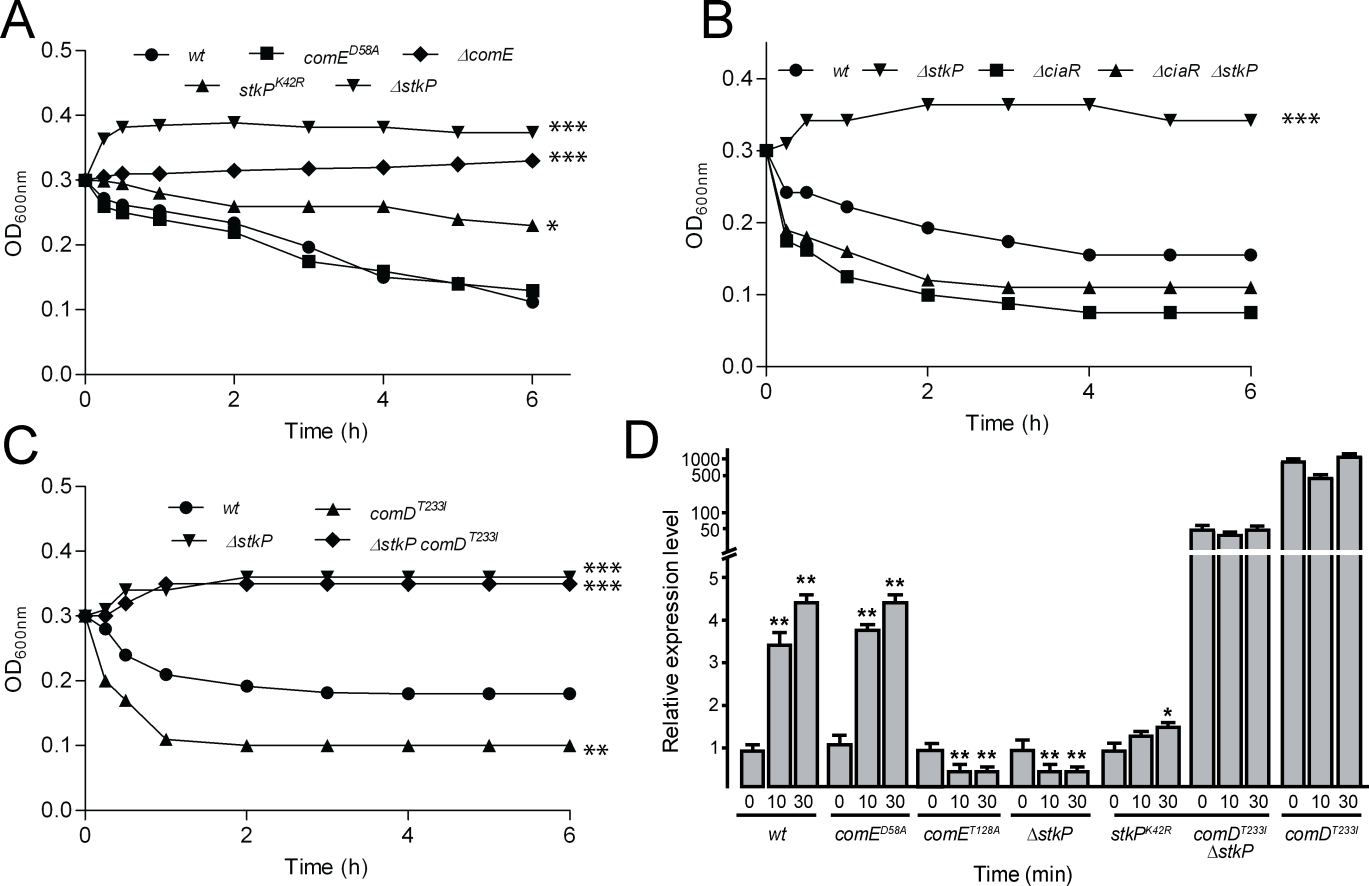
Evaluation of ASIL and *comE* expression in *S*. *pneumoniae* mutants. Autolysis was measured as a change in OD_620nm_ over 6 hours. Lytic curves corresponding to specific mutants are indicated in each panel (A-C), with data being representative of at least three independent experiments. (A) ASIL is controlled by StkP but it does not require Asp^58^-phosphorylation in ComE. (B) StkP does not participate in the CiaRH-regulated ASIL pathway. (C) StkP is involved in the ComE-regulated ASIL pathway. References: *p< 0.05; **p< 0.01; ***p< 0.001, these *p*-values were referred to the *wt* strain in each panels. (D) Transcription levels of the *comE* gene measured in cells exposed to pH 6.0. To avoid autolysis, all mutants were constructed in a Δ*lytA* (autolysin deficient) background. The Δ*lytA*, *comE*^*D58A*^ Δ*lytA*, Δ*stkP ΔlytA*, *comD*^*T233I*^ *ΔlytA and comE*^*T128A*^ *ΔlytA* cells were grown in ABM/pH 7.8 to the mid-exponential phase and resuspended in ABM/pH 6.0. Total RNA was extracted at 0 min, 10 min, and 30 min. The fold change in gene expression was measured by quantitative real-time PCR and calculated using the 2^–ΔΔCT^ method. The *gyrB* gene was used as the internal control and the reference condition was time 0 min of strain Δ*lytA*. Error bars indicate the standard deviation of the mean. INSTAT software was used to perform Dunnet’s statistical comparison test for each strain with its respective basal condition (time 0 min). References: **p< 0.01; ***p< 0.001.

Although ASIL is controlled by ComE activation without HK participation, this finding does not exclude the potential that ComE could have been phosphorylated by another phosphodonor at the Asp^58^ residue, typically the target of ComD phosphorylation [18]. Prokaryotic response regulators can be phosphorylated in vivo by acetyl-phosphate at the conserved aspartate residue of the receiver domain, resulting in similar activation to that exerted by the cognate HK [37]. In addition, phosphorylation crosstalk between HK and RR that belong to different TCSs has been reported [38]. Therefore, we first analyzed the possibility that Asp^58^ phosphorylation could be required for ASIL, and tested the *comE*^*D58A*^ mutant that encodes for the ComE^D58A^ protein, in which the phosphorylatable Asp^58^ residue is replaced by alanine [18,33]. The presence of this mutation was phenotypically corroborated under competence development conditions, confirming that CSP-induced competence was eliminated in the *comE*^*D58A*^ mutant (S1 Fig and [18]). Like the *wt* R801 strain, the *comE*^*D58A*^ mutant autolysed under acidic conditions (Fig 1A) indicating that Asp^58^ phosphorylation is not necessary for ASIL. The c*omE*^*D58A*^ phenotype is similar to the phenotype displayed by the *hk* mutants. Taken together, these results suggest ComE activation under acidic conditions is independent of both Asp^58^ phosphorylation and HK activity.

### The serine/threonine kinase StkP is essential for ASIL activation and participates in the ComE pathway

Since it has been shown that StkP is involved in *comCDE* expression during competence development at pH 7.8 [4], and that ComE is indispensable for the induction of ASIL [34], we evaluated whether StkP participated in ASIL development. Thus, the Δ*stkP* mutant strain did not autolyze when cultured under acidic conditions (Fig 1A), strongly suggesting that StkP is necessary for ASIL induction. To further confirm whether StkP kinase activity was required for ASIL, we also constructed the *stkP*^*K42R*^ mutant, which encodes for StkP with reduced enzymatic activity, as previously described [39]. This reduced kinase activity produces multiple septa, peripheral peptidoglycan biosynthesis and elongated cells [8], and these alterations were confirmed in our mutant and compared with the *wt* strain (S2A and S2B Fig). As expected, the *stkP*^*K42R*^ mutant strain showed ASIL with a degree of autolysis inferior to the *wt* strains but higher than the Δ*stkP* strain, indicating that the residual kinase activity in the *stkP*^*K42R*^ mutant [39] was likely responsible for ASIL induction. These observations strongly suggest that StkP kinase activity is essential for ASIL activation.

To exclude the possibility that the ASIL blockage observed in the Δ*stkP* mutant is a side effect due to hampered cell division and/or compromised cell wall structure [13], we constructed another mutant that presents cell division alterations, such as the Δ*mapZ* mutant [9]. We verified these alterations by Van-Fl staining (S2C Fig) and found that the Δ*mapZ* mutant showed an ASIL phenotype similar to the *wt* strain (S2D Fig), indicating that the ASIL effect showed by the Δ*stkP* mutant is independent of cell division alterations.

We have previously demonstrated that ASIL is controlled by two independent signaling pathways, CiaRH and ComE. While CiaRH plays a protective function, the ComE acts by promoting ASIL [34]. To determine whether StkP participated in the CiaRH-controlled ASIL pathway, the Δ*stkP* and Δ*ciaR* mutations were backcrossed, and the lytic phenotype of the resulting double mutants was analyzed. The Δ*stkP* Δ*ciaR* strain showed enhanced autolysis, similar to the Δ*ciaR* single mutant, demonstrating that the *ciaR* mutation had an epistatic effect on the non-autolytic *stkP* phenotype and that StkP activity did not participate in the signaling events of the CiaRH-controlled ASIL pathway (Fig 1B).

It has been previously described that the *comD*^*T233I*^ mutation produces a hyperactive ComD HK, which in turn hyperphosphorylates ComE, activates *comCDE* transcription, and results in high intracellular levels of ComE [40]. Although our results suggested that ComD was not involved in ComE-mediated ASIL, we constructed the *comD*^*T233I*^ mutant to artificially produce high levels of ComE and competence, as described [34,40]. The *comD*^*T233I*^ mutant showed constitutive high levels of *comE* transcripts, at 1000-fold higher compared to the *wt* strain. This mutant displayed accelerated ASIL compared to the *wt* strain, however, we previously reported that ASIL was blocked in the double *comD*^*T233I*^ *ΔcomE* double mutant [34]. These results demonstrated that ASIL induction was ComE-dependent and that increase expression of the latter leads to accelerated autolysis [34]. More importantly, when the *stkP* gene was disrupted in the *comD*^*T233I*^ mutant, ASIL was also completely blocked (Fig 1C), suggesting that StkP is essential for ASIL activation despite the high ComE levels expressed in the *comD*^*T233I*^ mutant.

### *comE* expression is induced in response to acidic conditions

It is known that *comE* is part of the *comCDE* operon, with the transcription of this operon initiated at the *pcomC* promoter (bp 2035421–2035806, *S*. *pneumoniae* R6 genome, NCBI reference: NC_003098.1) during competence development at alkaline pH [41]. To test whether *pcomC* was responsible for the increase in *comE* observed under acidic conditions, we constructed the *p*comC*-lacZ* reporter fusion (S1 Table), which was integrated via a single crossover event upstream of *comC* in a *bgaA* mutant (deficient in β-galactosidase activity). When the *bgaA* mutant strain carrying the *pcomC-lacZ* fusion was incubated at pH 6.0 for 30 min., a 1.7-fold increase in β-galactosidase activity was observed. No such increase was detected in a Δ*comE* knocked out mutant (S3 Fig). To further confirm this observation, the levels of *comE* transcript in the *wt* strain were determined by qPCR, which showed a 4-fold increase in cells exposed for 30 min at pH 6.0 (Fig 1D). These results indicate that the increased number of *comE* transcripts was caused by acidic stress, with this activation being dependent on ComE. Similarly, the levels of *comE* transcript in the *comE*^*D58A*^ mutant were increased 4.5-fold after 30 min incubation at pH 6.0. This result indicates that ComE^D58A^ was able to induce ASIL under acidic stress conditions (Fig 1D). Consequently, these results suggest that ComE is activated by an alternative signaling pathway that does not require phosphorylation of Asp^58^.

We previously reported that induction of *comE* transcripts by acidic stress is a characteristic of the ComE-mediated pathway that controls ASIL [34]. To examine whether StkP could be involved in this pathway, we analyzed the *comE* transcript levels by qPCR in the Δ*stkP* and *stkP*^*K42R*^ mutants constructed in a *lytA* background to avoid autolysis (S1 Table). After incubation of Δ*stkP* cells for 30 min at acidic pH 6.0, we observed a five-fold reduction in the levels of *comE* transcripts in the Δ*stkP* mutant (Fig 1D). In contrast, the *stkP*^*K42R*^ mutant showed a 2-fold decrease in *comE* transcripts, likely due to reduced kinase activity of StkP^K42R^. In addition, we observed that StkP was capable of controlling p*comC* activation by acidic stress since increased β-galactosidase activity was observed from the p*comC*-*lacZ* reporter fusion presence in the Δ*stkP* mutant in the *bgaA* background (S3 Fig). Taken together, these results indicate that StkP kinase activity was required for *comE* induction under acidic conditions.

In the *comD*^*T233I*^ *ΔstkP* double mutant, the levels of *comE* transcript increased 50 times over those in the Δ*stkP* strain and 10 times over those in the *wt* strain (Fig 1D). These results suggest that StkP kinase activity is required for full activation of ComE in order to induce ASIL under acidic conditions, regardless of the presence of high levels of ComE unnaturally induced by the ComD^T233I^ kinase. Such observations led us to speculate that StkP activates ComE by an alternative mechanism other than the classical ComD HK-mediated Asp^58^ phosphorylation.

### ComE is phosphorylated in vitro and in vivo by StkP

**Fig 2:**
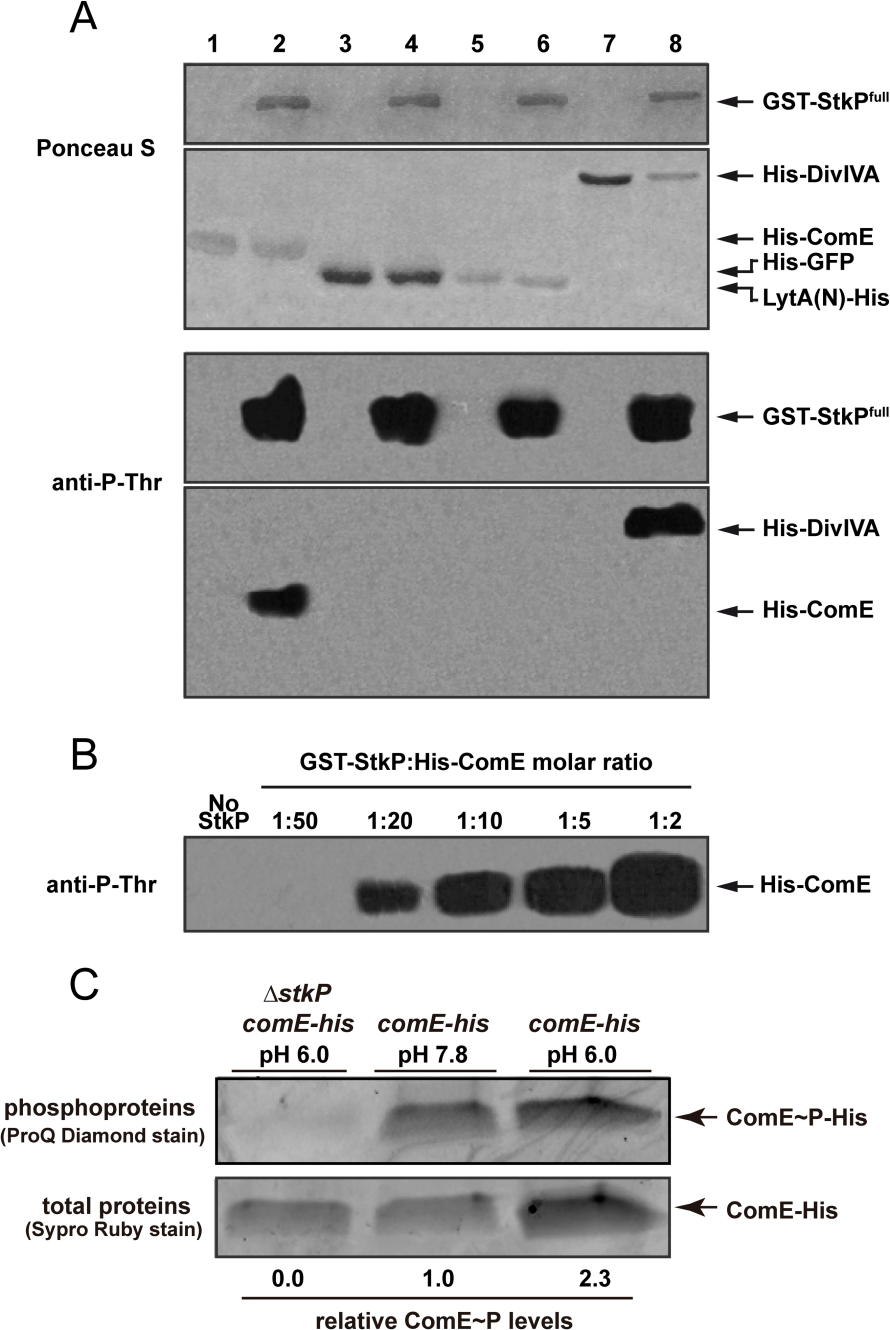
ComE is phosphorylated by StkP. (A) ComE is phosphorylated at a threonine residue by StkP. Top: nitrocellulose membrane stained with Ponceau S as a loading control. Bottom: Immunodetection of phosphorylated proteins. Phosphorylation reactions were carried out with purified GST-StkP and substrate proteins (0.5 μg each) mixed in kinase buffer and incubated at 37°C for 1 hour. Phosphorylated proteins were detected with an anti-phosphothreonine polyclonal antibody. Lane 1: His_x6_-ComE. Lane 2: His_x6_-ComE + GST-StkP. Lane 3: His_x6_-GFP. Lane 4: His_x6_-GFP + GST-StkP. Lane 5: LytA(N)-His_x6_. Lane 6: LytA(N)-His_x6_ + GST-StkP. Lane 7: His_x6_-DivIVA. Lane 8: His_x6_-DivIVA + GST-StkP. (B) ComE phosphorylation assays with different StkP:ComE molar ratios. GST-StkP and His_x6_-ComE were mixed at different molar ratios in kinase buffer and incubated at 37°C for 1 hour. Detection of phosphorylated proteins was performed as described above. (C) In vivo StkP-dependent and acid-induced ComE phosphorylation. C-terminal His-tagged ComE was purified from *wt* and *ΔstkP* strains grown in ABM (pH 7.8), and exposed to acidic stress in medium MD5, pH 6.0. Protein samples were separated by SDS-PAGE and phosphorylated or total ComE-His was detected with Pro-Q Diamond and SYPRO Ruby staining, respectively.

We hypothesized that StkP controls ComE by a crosstalk phosphorylation event. To test this hypothesis, we carried out an in vitro phosphorylation assay using purified recombinant His_x6_-ComE fusion protein, in the presence or absence of purified recombinant GST-StkP. The phosphorylation reactions were examined by immunoblotting using either anti-phosphoserine or anti-phospho-threonine antibodies. No signal was detected with the anti-phosphoserine antibody, in contrast, positive reactions were detected with the anti-phosphothreonine antibody (Fig 2A), with the phosphorylation reaction occurring at a molar ratio range of GST-StkP/His_x6_-ComE between 1:2 and 1:20 (Fig 2B). The GST-GFP or His_x6_-GFP were included as controls of reaction specificity, and His_x6_-DivIVA was used as a positive control of a StkP target, as previously described [8,11] (Fig 2A). We also evaluated the possibility that StkP could trigger ASIL by phosphorylating the major pneumococcal autolysin LytA. As overexpression of the full-length LytA protein was toxic in *E*. *coli* cells, the N-terminal region of LytA, which contains the catalytic domain was expressed instead fused to a His_x6_-tag (N-LytA-His_x6_)[42]). Incubation with or without GST-StkP, resulted in no evidence of N-LytA-His_x6_ phosphorylation (Fig 2A), suggesting that LytA was not phosphorylated by StkP, at least under the experimental conditions described here.

To determine whether StkP-mediated ComE phosphorylation occurs in vivo and because response regulators are usually expressed at low level in bacteria, we constructed by insertion-duplication mutagenesis *wt* and Δ*stkP* strain derivatives that express ComE fused to the His_x6_-epitope tag at the C-terminus (ComE-His_6x_) to improve ComE detection. Cells were incubated at either pH 7.8 or pH 6.0 and ComE-His_x6_ was purified from protein lysates as described in Material and Methods and separated by SDS-PAGE. Phosphoproteins were detected by ProQ Diamond staining while total proteins were detected by SYPRO Ruby staining. We observed that phosphorylated form of ComE in *wt* cells grown at pH 7.8, that increases 2.3 times when cells are grown at pH 6.0 (Fig 2C). In contrast, ComE remained unphosphorylated in the Δ*stkP* mutant, confirming that ComE is phosphorylated by StkP in vivo.

### StkP phosphorylates ComE at the Thr^128^ residue to regulate ASIL

**Fig 3:**
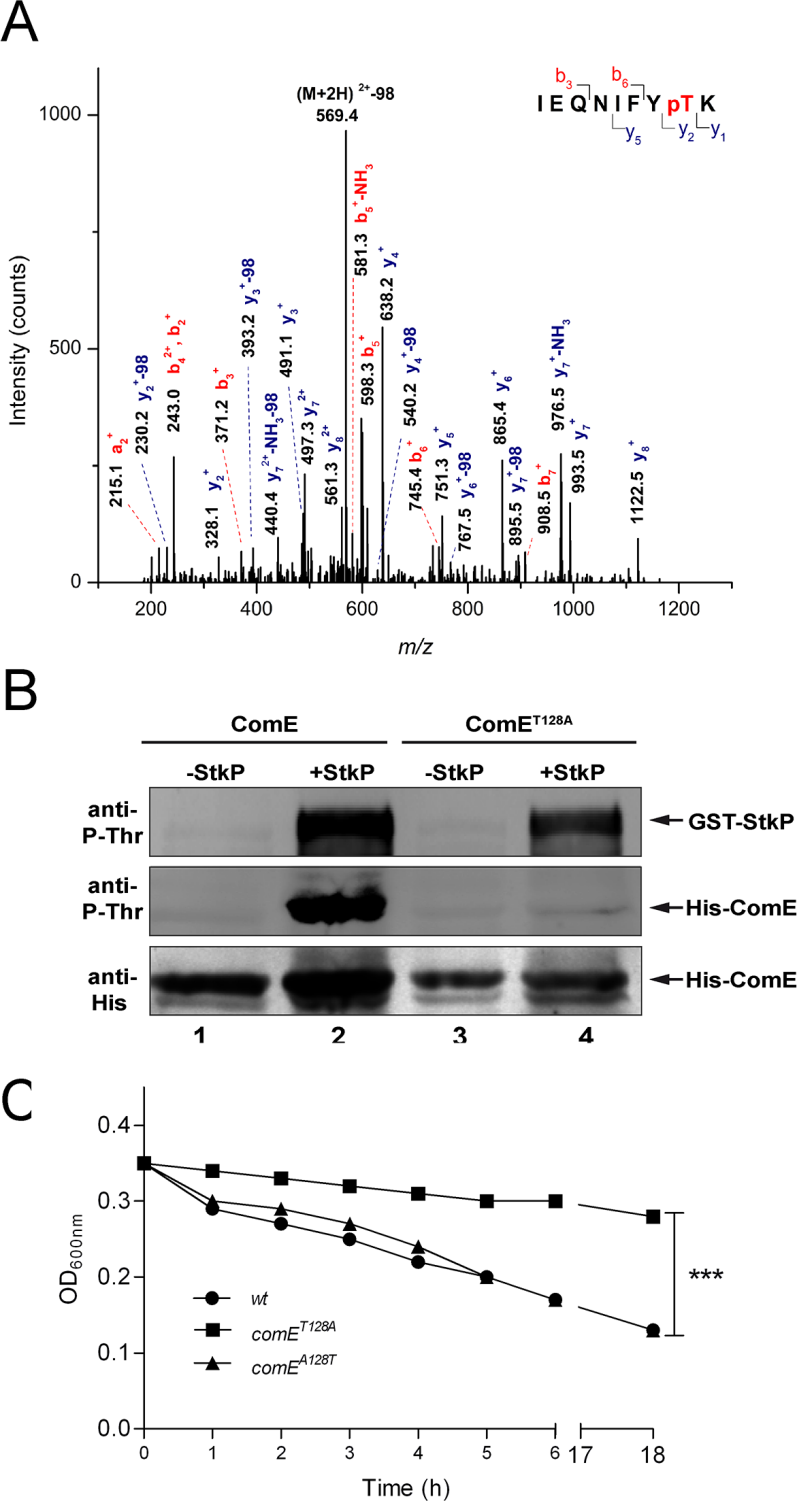
StkP phosphorylates ComE on the Thr^128^ residue to control ASIL. (A) To identify the phosphorylation site, tryptic peptides obtained from ComE previously incubated with StkP were analyzed by nano-LC-MS/MS. The figure shows the MS/MS spectrum of the di-charged ion of m/z 618.8 corresponding to the phosphorylated sequence IEQNIFYTK. C-terminal *y i*ons are labeled in blue, while N-terminal *a* or *b* fragment ions are labeled in red. Ions containing pT residue present the phosphorylation characteristic neutral loss of 98 Da. Thr^128^ is unequivocally identified as the phosphorylated residue (Xcorr 3, 45; pRS score 148). (B) StkP phosphorylates ComE at Thr^128^. *In vitro* phosphorylation assays were performed with purified GST-StkP and His_x6_-ComE^wt^ or His_x6_-ComE^T128A^ proteins mixed in kinase buffer at a StkP/ComE ratio of 1:20. Phosphorylated proteins were detected with an anti-phospho-threonine polyclonal antibody. Lane 1: His_x6_-ComE. Lane 2: His_x6_-ComE + GST-StkP. Lane 3: His_x6_-ComE^T128A^. Lane 4: His_x6_-ComE^T128A^ + GST-StkP. (C) ASIL requires the Thr^128^ residue in ComE for lysis induction. Autolysis was determined as indicated in the legend of Fig 1. Lytic curves corresponding to specific mutants are indicated, which data is representative of at least three independent experiments. References: ***p> 0.001.

To determine the amino acid residues in ComE that are targeted for phosphorylation by StkP, we performed HPLC-MS/MS analysis of the in vitro StkP-phosphorylated ComE-His_6x_ recombinant protein. A single amino acid was identified as a target for StkP-mediated phosphorylation in His_6x_-ComE, Thr^128^, located inside the trypsin-digested ^121^IEQNIFYTK^129^ ComE peptide (Fig 3A). To further confirm this observation, we created the non-phosphorylatable ComE^T128A^-His_6x_ recombinant mutant protein that remained unphosphorylated in the presence StkP in vitro (Fig 3B). To evaluate the role of Thr^128^ phosphorylation on ComE activity in vivo, we constructed the *comE*^*T128A*^ mutant, which showed significantly blocked autolysis compared to the *wt* (Fig 3C). Using the *comE*^*T128A*^ mutant, we produced the revertant *comE*^*A128T*^ strain, which showed an ASIL phenotype similar to the *wt* strain (Fig 3C). To further support the role of ComE Thr^128^ phosphorylation in ASIL activation, we attempted to replace Thr^128^ by Glu^128^ to construct the phosphomimetic *comE*^*T128E*^ protein, which is typically used to mimic the phosphorylated form of Thr residues [43]. In vitro, the phosphomimetic ComE^T128E^-His_x6_ protein was hyper-activated, as demonstrated by EMSA assays (see next), which may explain our inability to produce a viable *comE*^*T128E*^ mutant. These assays confirmed that Thr^128^ phosphorylation is essential for the StkP-mediated ComE activation that controls ASIL and that ComE hyper-activation is likely lethal to *S*. *pneumoniae*.

### Competence regulation is independent of Thr^128^ phosphorylation in ComE

StkP is involved in competence in response to stress conditions such as pH 7.8 [2,4,7]. Since, our results indicated that a signaling pathway that involves StkP and ComE controls autolysis in response to acidic stress at pH 6, we investigated whether this crosstalk mechanism could also regulate competence development at pH 7.8. We have previously described that the *stkP* mutant showed no competence development at pH 7.8 [4]. In contrast, the *comD*^*T233I*^ mutant shows a hypercompetent phenotype, accompanied by constitutively high levels of ComE expression [34]. We observed that the competence phenotype of the *comD*^*T233I*^ *ΔstkP* double mutant was similar to the *comD*^*T233I*^ single mutant (S1 Fig), indicating an epistatic effect of the *comD*^*T233I*^ mutation on the Δ*stkP* mutation. We also observed that the competence of the non-phosphorylatable *comE*^*T128A*^ and revertant *comE*^*A128T*^ mutants were similar to the *wt* strain (S1 Fig).

These observations suggest that StkP regulates competence at an early stage, which is independent of ComE Thr^128^ phosphorylation. Furthermore, StkP was not essential for competence once ComE was activated by ComD at pH 7.8. In contrast, StkP is necessary to activate ComE and to trigger autolysis under acidic conditions.

### StkP-mediated Thr^128^ phosphorylation increases ComE dimerization

**Fig 4:**
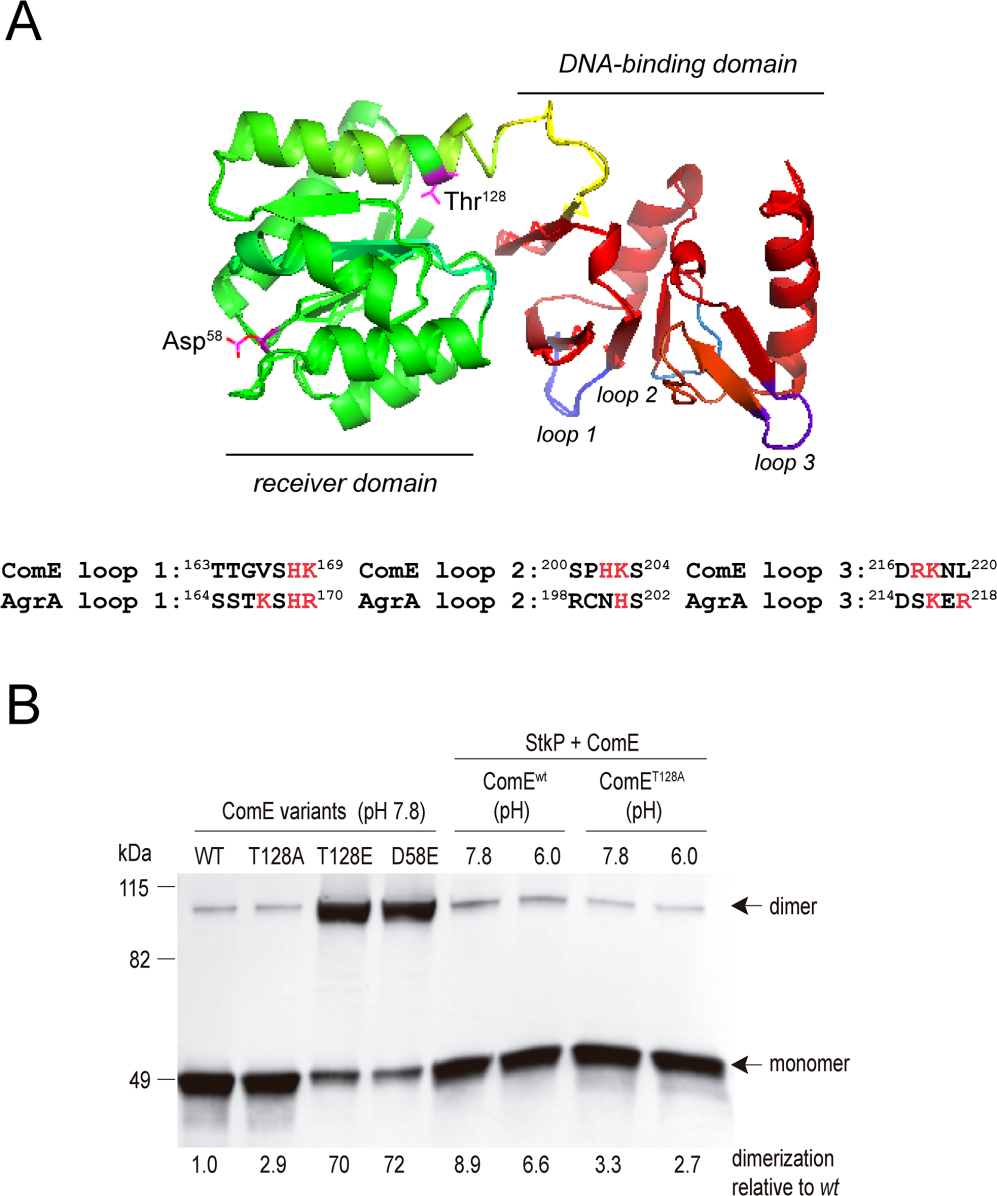
Thr^128^-phosphorylation increases the dimeric state of ComE. (A) Localization of Thr^128^ residue in the ComE structure. Based on the crystal structure of ComE reported by Boudes *et al* [43], this figure reveals the localization of the Thr^128^ residue, as well as the alternative phosphorylation site Asp^58^. The three loops in the DNA-binding domain are also shown, which are apparently altered when ComE is phosphorylated on Thr^128^. At the bottom of this image, a sequence alignment between the DNA-binding domains of ComE and AgrA is also shown. Positively charged or polar residues, which are described in AgrA to have a direct contact with DNA bases [45], are indicated in red. (B) The dimerization capacity of recombinant ComE proteins, such as the phosphomimetic ComE^D58E^ and ComE^T128E^ proteins, as well as the non-phosphorylatable ComE^T128A^ mutant, was analyzed and compared with ComE^wt^ (left panel). ComE^wt^ and ComE^T128A^ were also pre-incubated with GST-StkP (right panel). Dimerization states were assessed by native PAGE/Tris-MOPS buffer. Proteins were electroblotted onto PVDF membranes, and His_x6_-ComE was detected using anti-His antibody.

Response regulators are composed by a conserved receiver domain, which is phosphorylated on an aspartate residue by their cognate histidine kinase, and DNA-binding domains [44]. In ComE, the receiver domain corresponds to the first 130 residues [43]. Thr^128^ is located at the end of the α-5 region (Asp^114^-Ser^130^) of the receiver domain of ComE, next to the α-4 region (Ala^94^-Gln^101^), and near the loop between α-4 and β-5 (Val^102^-Leu^105^) that is involved in ComE dimerization and considered as a dimerization interface [43] (Fig 4A). The proximity between the dimerization interface and the two phosphorylated residues (Asp^58^ and Thr^128^) suggest a putative influence on the dimerization capacity of ComE, which was confirmed by in vitro dimerization assays using the phosphomimetic mutants. The ComE^D58E^-His_6x_, ComE^T128E^-His_6x,_ and ComE^T128A^-His_6x_ mutants showed dimer steady-state levels, which were 70, 72 and 2.9 times higher, respectively, than ComE^wt^-His_6x_ (reference level). In addition, when the ComE^wt^-His_6x_ protein was incubated with StkP, the dimerization rate increased 8.9 times, whereas ComE^T128A^-His_6x_ showed only a 3.3-fold increase (Fig 4B). The different dimerization capabilities found for ComE^wt^/StkP (8.9 times) compared to the phosphomimetic ComE^T128E^-His_6x_ (70 times) suggest that ComE^wt^-His_6x_ is partially phosphorylated by StkP (~12%). These observations suggest that Thr^128^ phosphorylation modifies ComE in a manner that strongly affects its dimerization interface, which is a condition *sine qua non* for the response regulator activation.

### Thr^128^ phosphorylation changes conformation of the ComE DNA-binding domain

In order to determine how Thr^128^ phosphorylation affected ComE's conformation, we performed molecular dynamic simulations at 40–150 ns comparing ComE^wt^ (PDB ID: 4CBV, [43]) and the in silico phosphomimetic ComE^T128E^-His_6x_ protein (S4 Fig, video). The simulations clearly indicated that 3 loops spanning the DNA-binding domain (Lys^218^-Asn^219^-Leu^220^, Thr^164^-Gly^165^-Val^166^-Ser^167^-His^168^, and Ser^200^-Pro^201^-His^202^-Lys^203^) presented different dynamics in the ComE^T128^ mutant compared to ComE^wt^ (Fig 4B and accompanying video shown in S4 Fig). ComE is a member of the AgrA/LytTR family of bacterial response regulators, which present certain structural homologies. Coincidently, two of these three loops have been described for the AgrA RR in *S*. *aureus* [45], as key residues that determine the DNA binding affinity for promoters in the phosphorylated form of AgrA (Fig 4B). ComE also revealed positively charged or polar residues (His^168^ and Lys^169^ in loop 1; His^202^ and Lys^203^ in loop 2; Arg^217^ and Lys^218^ in loop 3), which are shown in AgrA to have a direct contact with DNA bases [45]. These results suggest that after Thr^128^ phosphorylation ComE may undergo conformational changes. Consistent with this notion, limited proteolysis assays revealed structural differences between ComE-His_6x_ and ComE^T128E^-His_6x_ (S5 Fig). Treatment with trypsin showed more contrasting proteolytic patterns than proteinase K treatment. These findings confirmed that Thr^128^ phosphorylation causes evident changes in ComE conformation, likely in the DNA-binding domain of ComE, that may modify its DNA-binding affinity, and we explored this possibility with electrophoretic mobility shift assays (EMSAs).

### StkP-mediated Thr^128^ phosphorylation in ComE increases its DNA-binding affinity in a pH-dependent manner

**Fig 5:**
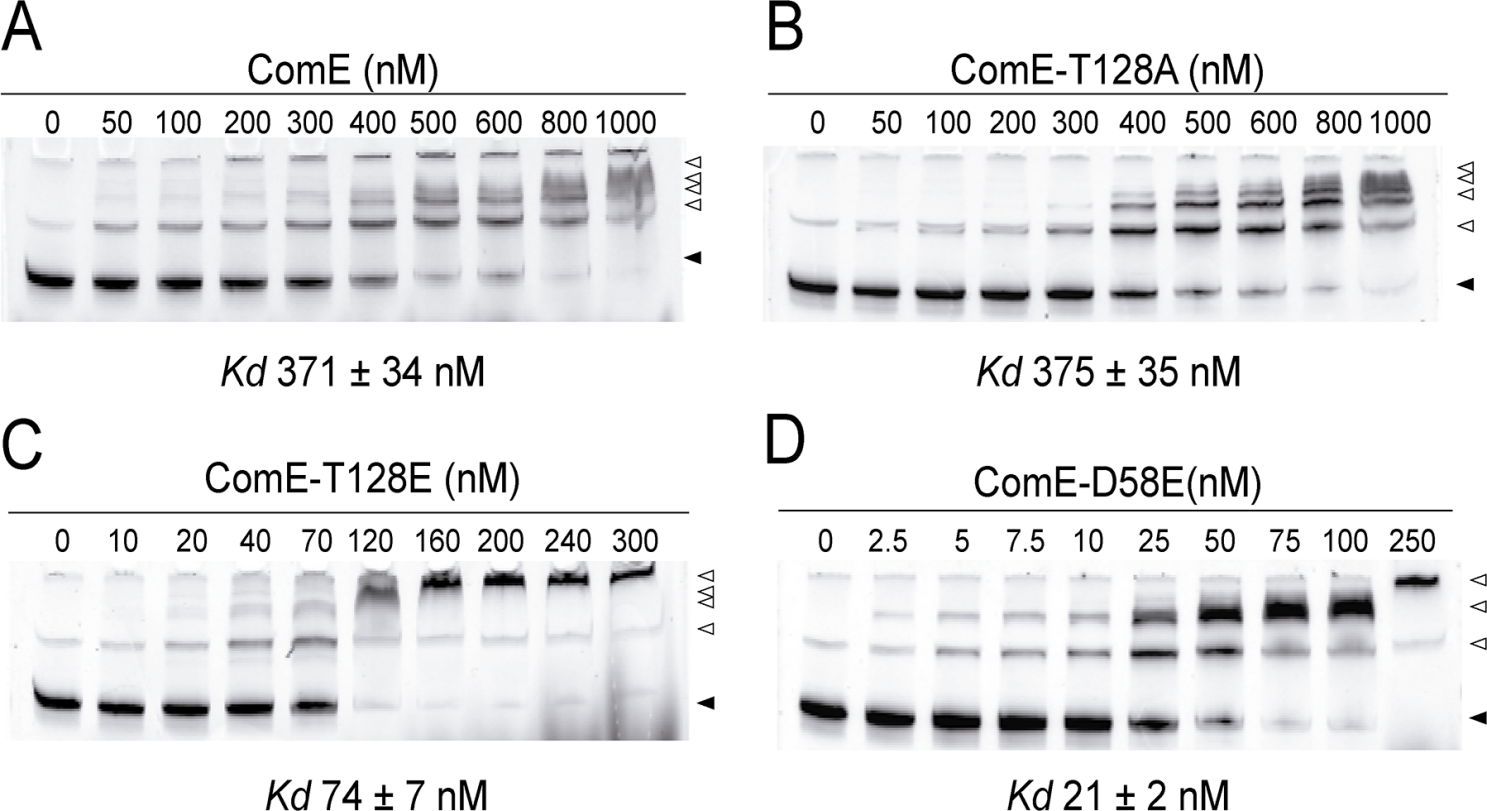
The phosphomimetic ComE^T128E^ protein shows an increased DNA-binding affinity. The DNA-binding affinity for the promoter region of the *comCDE* operon (p*comC*) of ComE^wt^ (A), the non-phosphorylatable (by StkP) ComET^128A^ mutant (B) and the phosphomimetic ComE^T128E^ (C) and ComE^D58E^ (D) proteins was determined by EMSA. Binding interactions were examined by incubating variable amounts of the different ComE versions with Cy5-labeled p*comC*, followed by electrophoretic separation of the protein-DNA complexes. Black or white triangles are indicating the free or ComE-bound probe, respectively. Images were obtained with a fluorescence scanner as described in Materials and Methods. The *Kd* values are indicated in each panel.

During competence development at alkaline pH, Asp^58^ phosphorylation by ComD results in increased binding of ComE to *pcomC* and transcriptional activation of the *comCDE* operon [2,46,47]. We have observed that at acidic pH, *pcomC* activation and induction of *comE* transcription depended both on ComE and on StkP (Fig 1D and S3 Fig) suggesting that Thr^128^ phosphorylation by StkP influences the binding of ComE to *pcomC*. Electrophoretic mobility shift assays (EMSAs) proved that the phosphomimetic ComE^T128E^-His_6x_ protein bound *pcomC* 5-fold stronger than ComE^wt^-His_6x_ (*Kd* 74 nM vs *Kd* 371 nM, respectively). The DNA binding affinity of the non-phosphorylatable ComE^T128A^-His_6x_ mutant was unaffected (*Kd* 375 nM) whereas ComE^D58E^-His_6x_ affinity for *pcomC* was 17- fold greater that ComE^wt^-His_6x_ (Fig 5 and Table 1). Curiously, when ComE^wt^-His_6x_ was pre-incubated with StkP in phosphorylation buffer at pH 7.8 no binding to *pcomC* was observed (S6 Fig, Table 1). As ASIL is regulated by StkP-mediated phosphorylation of ComE Thr^128^ residue under acidic conditions, we tested if ComE DNA-binding affinity could be affected by pH. When ComE^wt^-His_6x_ was previously incubated with StkP at pH 6.0, its affinity for p*comC* increased (*Kd* 64 nM) and was similar to that shown by ComE^T128E^-His_6x_ (S6 Fig, Table 1). To determine which of these contrasting effects actually depended on StkP phosphorylation, similar assays were performed with an inactive StkP enzyme (StkP^K42M^) [8] and ComE^wt^-His_6x_.

**Table 1:** ComE binding affinities to p*comC*.

	ComE/p*comC* dissociation constants (nM)				
ComE Variant	- StkP/pH 6.0	StkP/pH 7.8	StkP/pH 6.0	StkP^K42M^ /pH 7.8	StkP^K42M^/pH 6.0
WT	371 ± 34	>1000	64 ± 9	>1000	300 ± 17
T128A	375 ± 35	315 ± 29	311 ± 28	383 ± 36	336 ± 31
T128E	74 ± 7	93 ± 9	104 ± 9	ND	ND
D58E	21 ± 2	>800	38 ± 4	ND	ND

Binding to *pcomC* was comparable in ComE^wt^-His_6x_ pre-treated with StkP^K42M^ at pH 6.0 and untreated ComE^wt^ (*Kd* 300 nM vs *Kd* 371 nM) but was still absent after incubation at pH 7.8 (S7 Fig, Table 1). These results indicate that StkP phosphorylation at pH 6.0 underlied the enhanced *pcomC*-binding affinity of ComE, but not the blocking of ComE-*pcomC* interaction observed at pH 7.8, which suggests that at a slightly alkaline pH, StkP makes a complex with ComE masking its DNA-binding sites. The DNA-binding affinity of the phosphomimetic ComE^T128E^-His_6x_ and the non-phosphorylatable ComE^T128A^-His_6x_ proteins were not affected when preincubated with StkP at either pH 6.0 or pH 7.8 (S7 Fig, Table 1), indicating that Thr^128^ mediated the observed StkP effects on ComE: (1) at pH 6.0, Thr^128^ phosphorylation by StkP increases ComE DNA binding affinity; (2) at pH 7.8 Thr^128^ mediates the StkP-ComE interaction that blocks DNA binding. Thus, these experiments indicate that pH modulates the interplay between StkP and ComE.

### The StkP/ComE interaction increases at acidic pH

To test for a putative protein-protein interaction between StkP and ComE, a sandwich fluorescence-linked immunosorbent assay (FLISA) was utilized, in which the StkP-coated surface of a microtiter plate was incubated with increasing amounts of His-tagged ComE at either pH 6.0 or pH 7.8. This assay clearly showed that acidic pH augmented the number of binding sites between ComE and StkP, as reflected by a 3-fold increase in F_max_ (maximum fluorescence when binding is saturated) at pH 6.0 compared to pH 7.8 (Table 2, S8 Fig). The interaction between ComE^wt^ and the StkP^K42M^ mutant revealed the same F_max_ as the ComE/StkP at different pH values, indicating that kinase activity did not alter the saturation of this protein complex. However, for the StkP^K42M^ mutant, the *K*_*1/2*_ values were lower, indicating that StkP^K42M^ bound more tightly to ComE at any pH value. In comparison with ComE^wt^, the interaction between non-phosphorylatable ComE^T128A^ mutant and StkP or StkP^K42M^ was 3-fold stronger and was not affected by pH. In contrast, the phosphomimetic ComE^T128E^ protein produced a 3-fold increment in *K*_*1/2*_ at pH 7.8, which further raised to 9-fold at pH 6.0, demonstrating that the phosphorylated form of ComE had a lower affinity for StkP (Table 2, S8 Fig).

**Table 2:** Estimates of ComE binding affinities for StkP and StkP^K42M^.

		Binding to StkP^(a)^		Binding to StkP^K42M^^(a)^	
ComE variants	pH	*K*_*1/2*_ (ng)^(b)^	F_max_^(c)^	*K*_*1/2*_ (ng)^(b)^	F_max_^(c)^
WT	7.8	330 ± 60^(d)^	4700 ± 300	140 ± 20	4600 ± 200
WT	6.0	94 ± 8	11000 ± 200	55 ± 3	11800 ± 200
T128A	7.8	110 ± 20	4800 ± 300	80 ± 10	5000 ± 200
T128A	6.0	100 ± 10	11100 ± 400	100 ± 20	10800 ± 400
T128E	7.8	900 ± 100	5300 ± 600	ND^(e)^	ND
T128E	6.0	3200 ± 500	13300 ± 800	ND	ND

a) Fluorescence data was fit to F = F_max_ [ng ComE/(*K*_*1/2*_ + ng ComE)]

b)*K*_*1/2*_ is the amount of ComE (ng) required to reach half F_max_

c) F_max_ is the maximum fluorescence when the binding is saturated

d) Standard error calculated from at least 3 independent experiments

e) Not determined (ND)

These data confirm that the StkP/ComE interactions are mediated by ComE residue Thr^128^ and favored by acidic conditions, which may facilitate ComE phosphorylation.

### ComE induces global changes in the transcriptome of *S*. *pneumoniae*

**Fig 6:**
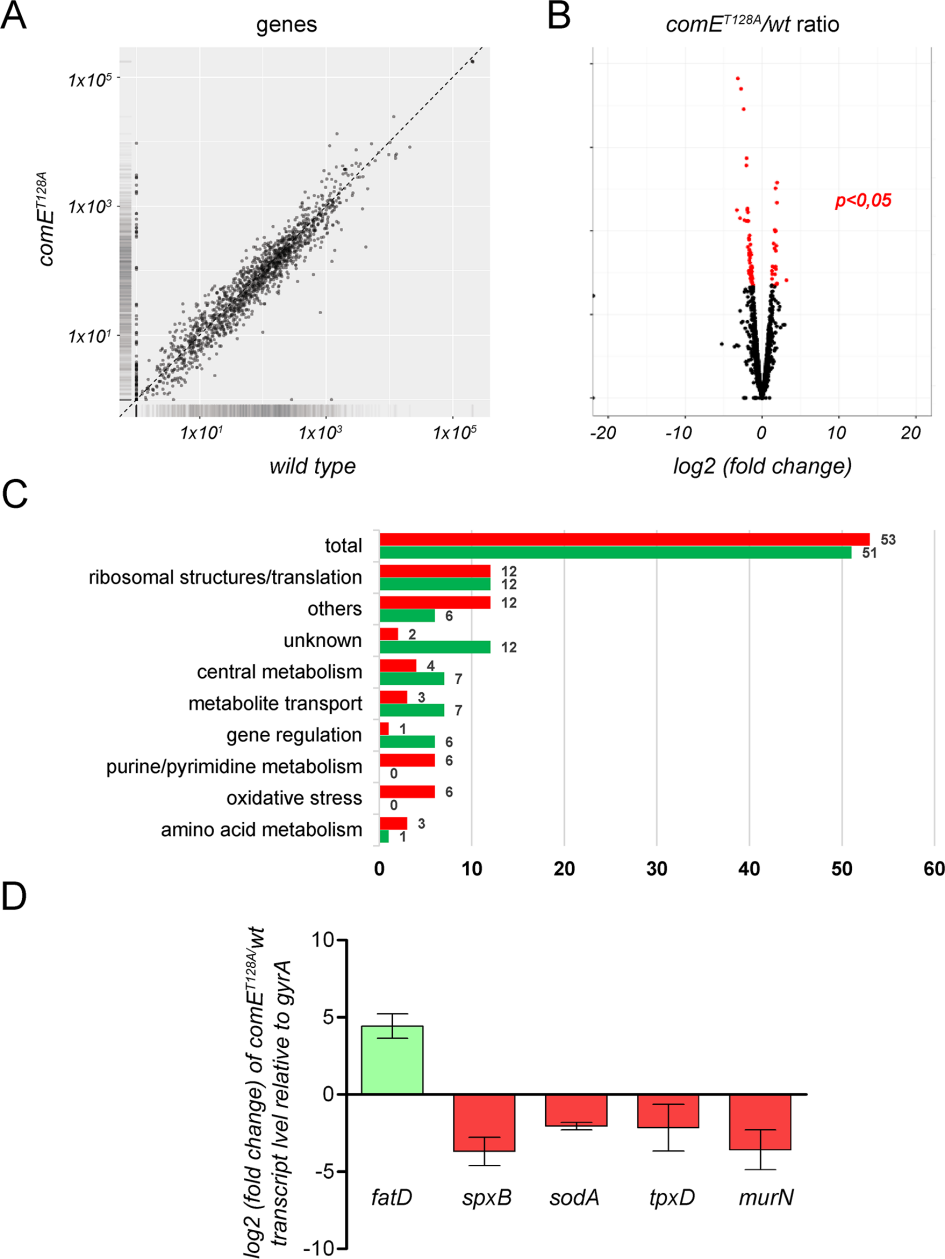
ComE is a global regulator that controls gene expression during the stress response. (A) Gene expression scatter plot in the *wt* and *comE*^*T128A*^ samples, with the *x*-axis representing the gene expression values for the control condition (*wt*) and the *y*-axis representing those for the treated condition (*comE*^*T128A*^). Each black dot represents a significant single transcript, with the vertical position of each gene representing its expression level in the experimental conditions and the horizontal one representing its control strength. Thus, genes that fall above the diagonal are over-expressed whereas genes that fall below the diagonal are underexpressed as compared to their median expression levels in the experimental groups. (B) Volcano plot of gene expression in *wt* vs *comE*^*T128A*^ samples measured by RNAseq. The *y*-axis represents the mean expression value of the log_10_ (*p*-value), while the *x*-axis displays the log_2_ fold change value. Black dots represent genes with an expression 2-fold higher in the *comE*^*T128A*^ mutant relative to strain *wt* with a *p*-value < 0.05, with red dots signifying genes with an expression 2-fold lower in the *comE*^*T128A*^ mutant, which are relative to strain *wt* with a *p* < 0.05. (C) Categories of ComE-regulated genes obtained from an RNAseq analysis. An RNAseq generated distribution in functional categories of genes that are regulated in the *comE*^*T128A*^ mutant relative to strain *wt* under acidic conditions. (D) ComE-regulated genes expressed under acidic conditions in the *comE*^*T128A*^ mutant relative to strain *wt*. Gene expression determined by RNAseq was confirmed by qPCR. The *comE*^*T128A*^ Δ*lytA* and Δ*lytA* (referred as *wt* for this assay) strains were grown in ABM/pH 6.0 to the mid-exponential phase in triplicate, with the fold change in gene expression measured by RT-qPCR and calculated using the 2^–ΔΔCT^ method. The *gyrA* gene was used as the internal control. References: ** *p* < 0.01; *** *p* < 0.001.

To understand the effect of the StkP/ComE signaling pathway on pneumococcal physiology, we compared the transcriptomes of the *comE*^*T128A*^ mutant and *wt* by RNAseq analysis. Three replicates of each strain strains were grown in ABM (pH 6.0) for 1 hr at the exponential growth phase (OD_620nm_ 0.3) and analyzed. In total, the differential expression of 104 genes was detected, 51 were down-regulated genes and 53 were up-regulated, considering relevant genes to be those with expressions higher than 2 fold and *p* values <0.05 (Fig 6A). The full list of these genes is shown (S3 Table). Based on this differential gene expression analysis, we observed that the StkP/ComE pathway affected, directly or indirectly, the expression of genes involved in oxidative stress, and the purine/pyrimidine, amino-acid and central metabolisms, as well as the ribosomal and translation structures, metabolite transport, molecular chaperones and cell wall biosynthesis, among others (Fig 6B). The list of genes regulated by Thr^128^-phosphorylated ComE indicated that this new signaling pathway induces global changes in the pneumococcal transcriptome, such as the physiological response to acidic stress. Bioinformatic analysis of the promoter regions (240 bp upstream of the start codon) of 22 ComE-regulated genes obtained from RNAseq assays predicted a putative DNA binding motif (S9A Fig). Martin *et al* [47] described a potential ComE^D58~P^ binding site (CEbs, 32 bp) in the *comC* promoter constituted by two repeats (DR1 and DR2) separated by 12 bp. In this report, we established that the putative ComE^T128~P^ binding site (26 bp) only partially overlaps with theses repeats suggesting a different consensus binding sequence to that described for ComE^D58~P^ (S9B Fig).

### The StkP/ComE pathway controls H_2_O_2_ production and oxidative stress response

**Fig 7:**
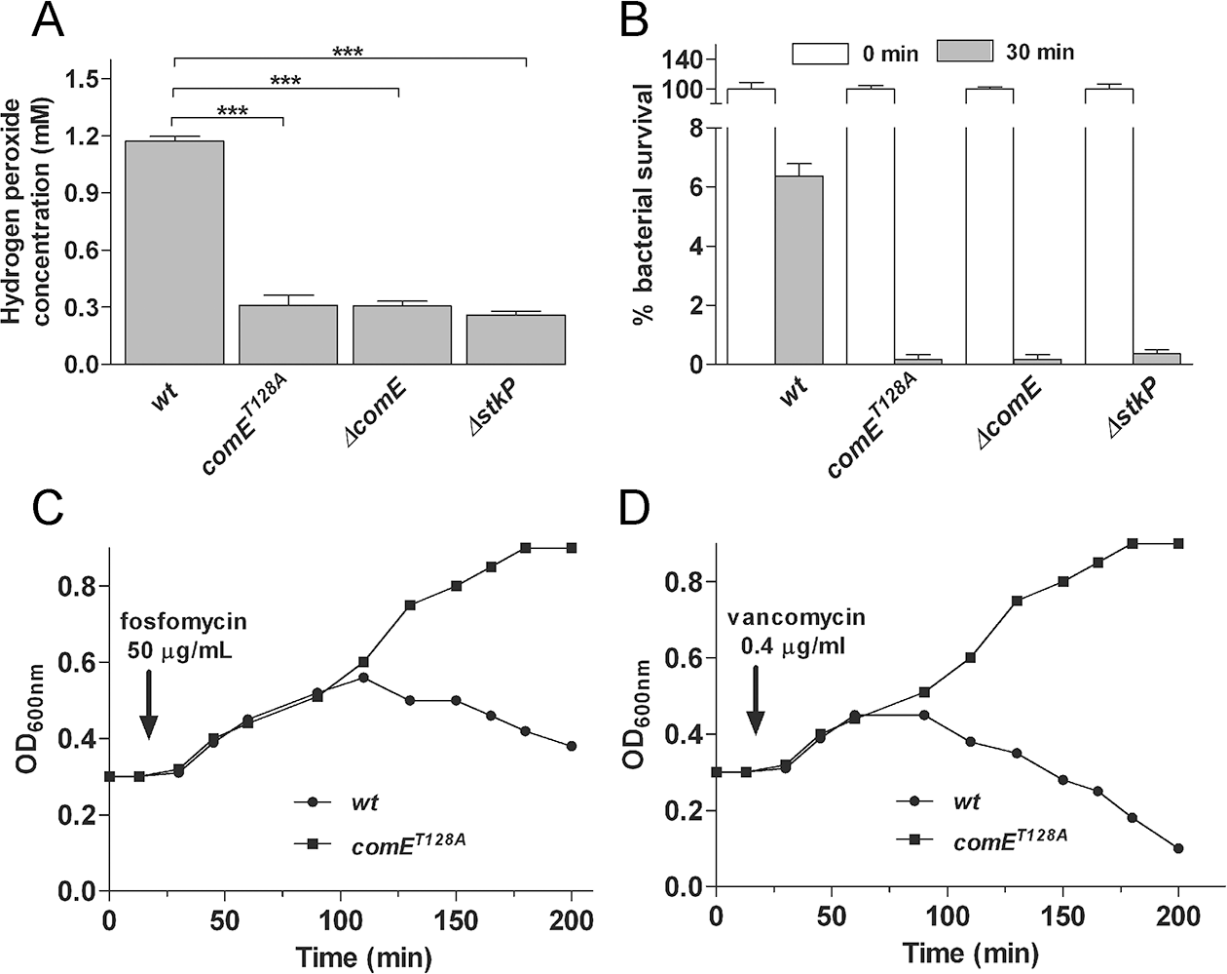
The StkP/ComE pathway controls oxidative stress and cell wall biosynthesis. **(**A) The H_2_O_2_ production is altered in the *comE* and *stkP* mutants. Cells were grown in BHI at 37°C to an OD_620nm_ of 0.3, then diluted in either ABM (pH 6.0) and incubated at 37°C to an OD_620nm_ of 0.3. The H_2_O_2_ concentration was determined by the peroxidase test as described in Material & Methods. Values were calculated as the H_2_O_2_ concentration in mM and normalized against the number of viable cells. (B) The *comE and stkP* mutants were more susceptible to H_2_O_2_ than *wt*. Susceptibility to H_2_O_2_ is indicated as a percentage of bacterial survival at different time points. C-D) The *comE*^*T128A*^ mutant was more resistant to cell-wall antibiotic-induced lysis than *wt*. Cells were grown in BHI/pH 7.2 at 37°C to an OD_620nm_ of 0.3, and fosfomycin (C) and vancomycin (D) were added in independent cultures at final concentrations of 50 μg/ml and 0.4 μg/ml, respectively. Cell lysis of bacterial cultures was determined by turbidimetry at OD_620nm_ for more than 3 h. References: *** p < 0.001.

Using RT-qPCR we confirmed decreased expression of oxidative stress genes *spxB*, *sodA* [48] and *tpxD* [49] in *comE*^*T128A*^ mutant compared to *wt* (Fig 6C). The *spxB* gene encodes the pyruvate oxidase that produces H_2_O_2_ from O_2_, *sodA* encodes the superoxide dismutase that produces H_2_O_2_ from superoxide, and *tpxD* encodes the thiol peroxidase that catalyzes the H_2_O_2_ oxidation and contributes to the oxidative stress response. In the *comE*^*T128A*^ mutant, we found that the *spxB*, *sodA*, *and tpxD* transcripts were downregulated 18, 2.8 and 2.7 times, respectively (Fig 6C). These findings were also corroborated by H_2_O_2_ production and H_2_O_2_ susceptibility assays. The *comE*^*T128A*^, *ΔcomE*, *and ΔstkP* mutants showed a 4-fold decrease in their H_2_O_2_ production compared to the *wt* and *comE*^*A128T*^strains (Fig 7A), which is likely caused by the reduced expression of *spxB* and *sodA* protein products. In addition, we observed a 10-fold reduction in the susceptibility to H_2_O_2_ by the *comE*^*T128A*^, *ΔcomE*, *and ΔstkP* mutants compared to the *wt* strain (Fig 7B), likely due to reduced expression of the TpxD peroxidase. These findings support the notion that the StkP/ComE pathway is essential for the control of H_2_O_2_ production and for H_2_O_2_ tolerance.

### The StkP/ComE pathway regulates *murN* expression and modulates susceptibility to antibiotic-induced lysis

Although RNAseq analysis showed that the *murN* gene was overexpressed in the *comE*^*T128A*^ mutant, its expression by qPCR was actually found to be 4-fold lower than in *wt* in three independent assays (Fig 6C), suggesting a typical case of false positive that is commonly found in RNAseq studies. Regarding the physiological impact of the *murN* mutation, Filipe *et al* [50] described that a *murMN* mutant had cell wall alterations and presented increased susceptibility to lysis when exposed to cell wall antibiotics. To test whether an altered *murN* expression in the *comE*^*T128A*^ mutant could modify the susceptibility to cell wall antibiotics, we determined the MIC values of the *comE*^*T128A*^ and *wt* strains in the presence of either fosfomycin, vancomycin, penicillin, cefotaxime, cefazolin, or piperacillin. The fosfomycin MIC in the *comE*^*T128A*^ (170 μg/ml) was higher than the *wt* strain (50 μg/ml), whereas the MICs for vancomycin, penicillin, cefotaxime, cefazolin, and piperacillin were similar between these strains. The typical lytic effect of fosfomycin (50 μg/ml, 1xMIC; Fig 7C) and vancomycin (0.4 μg/ml, 1xMIC; Fig 7D) on the *wt* strain was inhibited in the *comE*^*T128A*^ strain. The diminished susceptibility to cell wall antibiotics in the *comE*^*T128A*^ strain suggests cell wall alterations consistent with the ASIL repression showed by this mutant.

### Pneumococcal survival in pneumocytes is controlled by the StkP/ComE pathway

**Fig 8:**
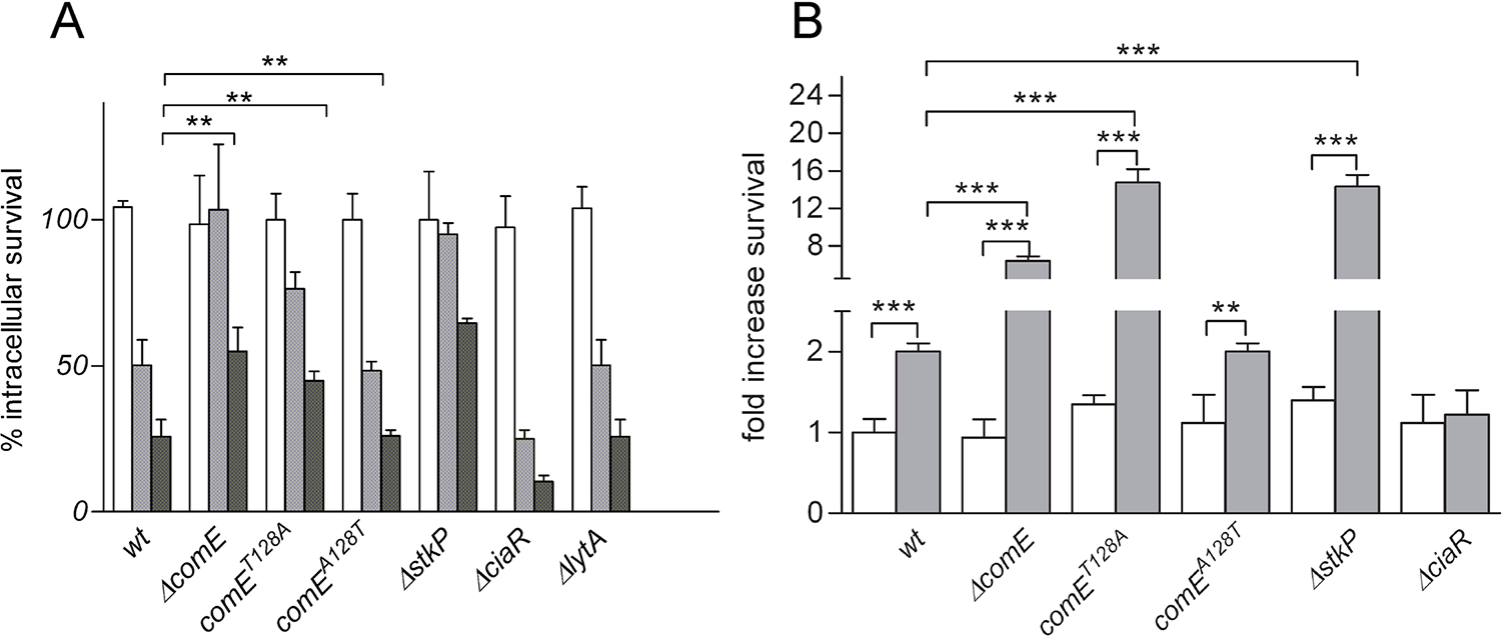
The StkP/ComE pathway modulates intracellular survival and the acid tolerance response of *S*. *pneumoniae*. (A) The Δ*stkP* and Δ*comE* mutants showed increased intracellular survival compared with *wt* in A549 pneumocytes. Bacteria cells were initially incubated for 3 h in monolayers of A549 pneumocytes, and survival progression of different strains was monitored using a typical protection assay. Survival percentages were calculated by considering the total amount of internalized bacteria after 30 min of extracellular antibiotic treatment as representing 100% for each strain. After antibiotic treatment, samples were taken at 0 (white bars), 3 (grey bars) and 7 (black bars) hours, and pneumocytes were lysed to release pneumococci. Samples were diluted in BHI spread on BHI-blood-agar plates and incubated at 37°C for 16 h. (B) The Δ*stkP* and Δ*comE* mutants displayed an augmented ATR compared with *wt*. To determine the survival percentage of bacterial strains, the non-induced cells (white bars) were directly exposed for 2 h at pH 4.4 (lethal pH) in THYE medium, with the acid-induced cells (grey bars) being previously incubated for 2 h at pH 6.0 (sub-lethal pH) in THYE medium. After exposition to lethal pH, pneumococcal survival was determined by spreading dilutions in BHI-blood-agar plates and incubating these at 37°C for 16 h. For both panels, data are representative of at least three independent experiments and statistically significant differences are indicated as *p*<0.01 (**) or *p*<0.001 (***).

We previously described that ComE is involved in the acidic stress response and in the pneumococcal intracellular survival mechanism in pneumocytes [33]. Here, we demonstrate that StkP phosphorylates ComE, and in order to determine whether this crosstalk affects the pneumococcal survival, we measured the intracellular survival capacities in A549 pneumocyte cells of the Δ*stkP*, *stkP*^*K42R*^ (reduced kinase activity), Δ*comE*, *comE*^*T128A*^ and *comE*^*A128T*^ (revertant) strains compared to the *wt* in A549 pneumocyte cells [33]. Mutations in either the *stkP* or *comE* genes conferred increased survival compared to *comE*^*A128T*^ or *wt* (Fig 8A), indicating that the StkP/ComE pathway controlled pneumococcal survival in pneumocytes. To discriminate whether this phenotype could result from increased ATR or decreased ASIL, we tested the Δ*lytA* mutant, which lacks autolysin and presented a blocked ASIL [33], but its intracellular survival was similar to the *wt* strain (Fig 8A). Thus, a blocked ASIL is not enough to increase intracellular survival of *S*. *pneumoniae* in pneumocytes. Consequently, the increased survival showed by either the Δ*stkP*, Δ*comE*, or *comE*^*T128A*^ mutants (Fig 8A) is likely due to higher ATR capacity. To test this hypothesis, we determined the ATR phenotype of the *comE*^*T128A*^ mutant, but in a *lytA* deficient background in order to discard residual autolysis. As expected, ATR of the Δ*lytA* strain increased 2-fold at pH 6.0 compared with cells cultured at pH 7.8, whereas the *comE*^*T128A*^ Δ*lytA* cells showed a 20-fold increase under the same conditions (Fig 8B), supporting the notion that increased ATR explains the increased survival rate displayed by the *comE*^*T128A*^ mutant in pneumocytes.

## Discussion

Two-component systems (TCSs) represent one of the most important mechanisms of gene regulation in bacteria. Alternatively, eukaryotic-like serine-threonine kinases (STKs) constitute another signaling mechanism that bacteria utilize to regulate different cellular functions, such as stress response and pathogenesis. STKs are more promiscuous than the TCS-associated kinases and can phosphorylate different protein substrates producing pleiotropic effects [16,51,52]. STKs are also able to interact with TCSs by direct phosphorylation of RRs, as reviewed in [3,53]. STK-mediated RR activation takes place on either serine or threonine residues, instead of aspartate, which is the typical residue target for HK phosphorylation. STK-mediated phosphorylation on DNA-binding domains of RR have been reported, as described for GraR in *S*. *aureus* [54], YvcK in *Listeria monocytogenes* [55] and RitR in *S*. *pneumoniae* [56]. STKs may also phosphorylate on receiver domains, as observed for CovR in *S*. *pyogenes* [57], WalR in *B*. *subtillis* [58], DosR in *M*. *tuberculosis* [59], or in both domains, as demonstrated for VraR in *S*. *aureus* [60].

ComE is the most studied RR in *S*. *pneumoniae* and belongs to the AlgR/AgrA/LytTR transcription factor subfamily, showing a typical receiver domain and a DNA-binding (or LytTR) domain. When phosphorylated by the ComD histidine kinase at the Asp^58^ residue, ComE undergoes conformational changes that increase their DNA affinity and modify transcription regulation of competence genes by binding to their promoter regions [2,47,61]. In the present work, we show that *S*. *pneumoniae* utilizes an alternative signal transduction pathway to control acidic stress response (ASIL and ATR), oxidative stress, cell wall biosynthesis, and intracellular survival in pneumocytes. Under acidic conditions, a phosphorylation crosstalk between StkP and ComE involving phosphorylation at Thr^128^ in the receiver domain resulted in activation of this RR.

Using the crystal structure of ComE [43], a molecular dynamic simulation of ComE permitted a comparison with the phosphomimetic ComE^T128E^ protein, predicting that the Thr^128^ phosphorylation produces structural changes in the DNA-binding domain. These putative conformational changes were confirmed by limited proteolysis assays that revealed differences between ComE and ComE^T128E^. In this sense, we observed that phosphorylation at either Asp^58^ or Thr^128^ increases the dimerization and DNA-binding capacity of ComE. These results were coincident with the activation model proposed by Boudes *et al* [43], where the most plausible activation mechanism of ComE is first a phosphorylation reaction to induce its dimerization, which occurs at the canonical receiver domain of ComE, followed by binding to DNA via the LytTR domain. It remains to be elucidated how phosphorylation at alternative sites in the receiver domain (Asp^58^ or Thr128) modifies the DNA-binding domain of ComE.

Because ComE Thr^128^ activation is independent of CSP/ComD activation by a quorum sensing mechanism, this RR requires alternative factors to act as a sensor and/or an environmental signal to trigger an adaptive stress response. We propose that StkP senses an alternative environmental signal, acidic pH. Our results are consistent with such notion: the level of *comE* transcripts induced under acidic conditions is the indicator of ComE activation due to the *comCDE* operon is autoregulated.

An additional aspect to consider in the StkP/ComE crosstalk phosphorylation is the effect of pH on protein-protein interactions. We observed that the non-phosphorylated forms of both proteins show strong interaction at acidic pH. Once StkP auto-phosphorylates, it becomes metastable complex until it dissociates from the phosphorylated ComE form. Such cycle is favored at pH 6.0 and evidence for the outcome of such cycle is shown by the fact that phosphorylation of ComE at Thr^128^ by StkP prevents further interaction between these two proteins. We propose the following cycle for StkP/ComE interaction:

StkP+ComE→StkP/ComE→StkP‑P/ComE→StkP+ComE‑P(stablecomplex)(transientcomplex)

Pneumococcal H_2_O_2_ production is one of the most significant among bacterial pathogens, and *S*. *pneumoniae* utilizes this intermediate metabolite to compete with the respiratory tract microbiota and to produce cytotoxic effects on the host tissues [62–64]. In this investigation, the transcriptome analysis of the *comE*^*T128A*^ mutant revealed a marked decrease in the expression of *spxB* and *sodA* when cells were grown under acidic conditions. These results correlated with very low H_2_O_2_ production by the *comE*^*T128A*^ mutant associated with low expression of SpxB and SodA. Considering that H_2_O_2_ is toxic for eukaryotic cells, we propose that reduction H_2_O_2_ production in the *comE*^*T128A*^ mutant facilitates its intracellular survival in pneumocytes. *S*. *pneumoniae* generates hyper-virulent mutants with defective *spxB* during infection [65], supporting the hypothesis that the H_2_O_2_ levels are controlled during pneumococcal pathogenesis.

Because *S*. *pneumoniae* lacks catalase, and H_2_O_2_ overproduction must be controlled for this pathogen to survive, this pathogen induces an oxidative stress resistance that is induced by endogenous H_2_O_2_ [66]. In this sense, the transcriptome analyses of the *comE*^*T128A*^ mutant revealed a decreased expression of *tpxD*, which encodes the thiol peroxidase. These findings correlate with the increased H_2_O_2_ susceptibility by the *comE*^*T128A*^ mutant. Similarly, a previous study showed that a *tpxD* (or *psaD*) mutant eliminated the H_2_O_2_-mediated response to high H_2_O_2_ levels [67]. Previously, a microarray analysis of the *stkP* mutant revealed that *tpxD* expression is repressed, which is correlated with a low H_2_O_2_ resistance of this mutant, but the putative regulatory mechanism was not mentioned [7]. In the present study, we have shown for the first time that the StkP/ComE pathway controls oxidative stress resistance and H_2_O_2_ production under acidic conditions, which are probably responsible for the intracellular survival of *S*. *pneumoniae* in pneumocytes.

In a previous study, we proposed that ASIL may be activated under acidic conditions by a translocation of LytA from an intracellular to an extracellular compartment probably due to cell wall alterations by an unknown ComE-dependent mechanism [68]. Here, the *comE*^*T128A*^ mutant displayed a decreased expression of *murN*, which encodes one of the first enzymes involved in the cell wall biosynthesis of *S*. *pneumoniae* [69]. Filipe *et al* [70] described that the *murMN* mutant showed an increased susceptibility to lysis when *murMN* cells were exposed to cell wall antibiotics, such as fosfomycin and vancomycin, which are involved in the inhibition of both the early and late stages of cell wall biosynthesis, respectively. Following this line of thinking, an altered expression of *murN* in the *comE*^*T128A*^ mutant should cause a misbalance in the peptidoglycan biosynthesis and modify susceptibility to cell wall antibiotics. Accordingly, we observed that this mutant had an increased MIC of fosfomycin compared with *wt*, as well as a greater tolerance to autolysis induced by fosfomycin or vancomycin. The putative cell wall alterations indicated by antibiotic susceptibility tests may explain the autolysis inhibition shown by the *comE*^*T128A*^ mutant under acidic stress, which probably interfered with LytA activation. Work is in progress to try to determine the nature of such cell wall alterations.

We also demonstrated that Thr^128^ phosphorylation is not involved in competence. Regarding this topic, Guiral *et al* [71] described a phenomenon of lysis of non-competent cells triggered by competent cells, named allolysis, which involves bacteriocins and the autolysins LytA, LytC, and CbpD. Allolysis is considered to be a competence-induced mechanism of predation of non-competent cells that contributes to virulence by releasing pneumolysin [72]. Here, we show that the StkP/ComE signaling pathway can also trigger autolysis of noncompetent cells in acidic biological niches, such as inflammatory foci or endosomal compartments. This phenomenon occurred without the activation of a quorum sensing mechanism, a situation that allows bacterial cells to lyse even under low population density conditions.

**Fig 9:**
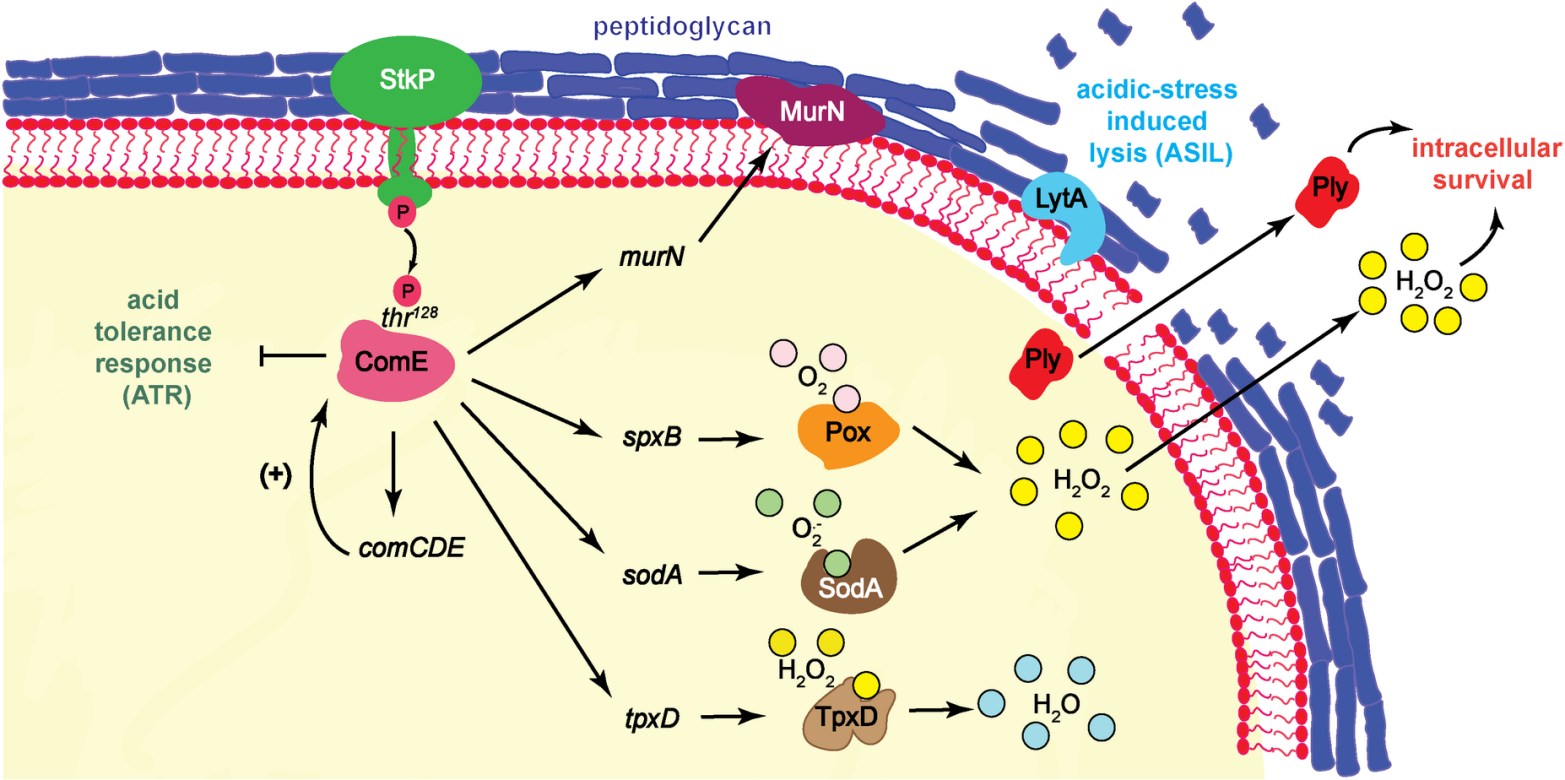
Proposed model for crosstalk between StkP and ComE that impacts on the acidic stress response and intracellular survival mechanisms in *S*. *pneumoniae* exposed to acidic conditions.

StkP and ComE have already been shown to be involved in pneumococcal pathogenesis in different studies using animal models, with StkP appearing to be involved in bacterial survival in vivo [4,73]. On the other hand, ComE-mediated competence for DNA transformation has been also associated with virulence [74,75]. As mentioned above, pneumolysin release by competence-mediated autolysis was considered to be essential for pneumococcal pathogenesis [71]. Concerning the impact of StkP/ComE pathway regulation on pneumococcal pathogenesis, we propose that two different scenarios should be considered. In extracellular niches, a subpopulation of pneumococci exposed to acidic stress may cause tissue damage by overproduction of H_2_O_2_ and induction of ASIL to release pneumolysin, with this suicidal situation being promoted by StkP-mediated phosphorylation of ComE. On the other hand, the Thr^128^-nonphosphorylated form of ComE might facilitate pneumococcal survival at either the extracellular or the intracellular level in host tissues. We focused our attention on the first barrier that this pathogen must cross to establish an infection, and we hypothesized that intracellular survival in pneumocytes should be important for *S*. *pneumoniae*. The Δ*stkP*, Δ*comE*, and *comE*^*T128A*^ mutants were tested in the pneumococcal infection model in A549 pneumocytes, and they revealed an increased survival compared with *wt*. Thus, we conclude that this survival could have been caused by increasing their capacity of ATR, decreasing the H_2_O_2_ production and modifying the cell wall biosynthesis to repress ASIL (Fig 9). Establishing how the balance between H_2_O_2_ resistance mechanism and H_2_O_2_ production affects intracellular survival is beyond the scope of this report, but this work is in progress. Finally, we propose that the StkP/ComE pathway is relevant in the genetic regulation of physiological adaptation to environmental stress, which is necessary for pneumococcal survival in pneumocytes. This is one of the first steps in the pathogenic process that *S*. *pneumoniae* must overcome to produce infection.

## Materials and methods

### Bacterial strains, plasmids, cell lines, and growth conditions

All strains, plasmids, and oligonucleotides used in this study, as well as cloning and mutagenesis procedures, are listed in the supplementary material (S1 Table). The growth conditions and stock preparation for the pneumococcal and *Escherichia coli* strains have been reported elsewhere [34], and the transformation assays have also been previously described [76,77].

### ASIL and ATR assays

ASIL was performed as described previously [33]. Firstly, bacterial cells were grown in Todd-Hewitt/yeast extract medium. When cultures reached OD_600nm_ ~0.3, cells were centrifuged at 10,000 g for 5 min, the pellet was resuspended in ABM pH 6.0 and cultures were re-incubated at 37°C. Autolysis was measured as a change in OD_600nm_ at different time points over 6 h.

ATR was performed as described previously [24]. For non-acid-induced conditions, bacterial cells were first grown in THYE (pH 7.8) at 37°C, and when cultures reached OD_600nm_~ 0.3, 100 μl aliquots were taken and added to 900 μl of THYE (pH 4.4) and incubated for 2 h at 37°C. Then, serial dilutions were made in THYE (pH 7.8) and plated onto 5% of sheep blood tryptic-soy agar (TSA) plates. After 24 h of incubation at 37°C, colonies were counted to determine the number of survivors, with the total CFU being obtained by plating serial dilutions of cells grown THYE pH 7.8 onto 5% sheep blood TSA, made just before cells were switched to pH 4.4. In parallel, to determine survival under acidic-induced conditions, bacterial cells were grown in THYE (pH 7.8) until OD_600nm_ ~ 0.3, centrifuged at 10,000 g for 5 min, resuspended in THYE (pH 6.0) and incubated for 2 h at 37°C. Culture aliquots were taken and serially diluted in THYE pH 7.8 for total cell counting, while other aliquots were diluted ten times in THYE (pH 4.4) and incubated for 2 h at 37°C to determine the survival percentage as described above. For both assays (acid-induced and non acid-induced conditions), this was calculated by dividing the number of survivors at pH 4.4 by the number of total cells at time zero (before incubation at pH 4.4). For the ASIL and ATR assays, data were expressed as the mean percentage ± standard deviation (SD) of independent experiments performed in triplicate.

### Cell lines and culture conditions

The A549 cell line (human lung epithelial carcinoma, pneumocytes type II; ATCC CCL-185) was cultured at 37°C, 5% CO_2_ in Dulbecco’s modified Eagle medium (DMEM) with 4.5 g/l of glucose and 10% of heat-inactivated fetal bovine serum (FBS)(Gibco BRL, Gaithersburg, Md.). Fully confluent A549 cells were split once every two or three days via trypsin/EDTA treatment and diluted in fresh media before being cultivated in Filter cap cell flasks of 75 cm^2^ (Greiner Bio-one no. 658175) until passage 6.

### In vitro phosphorylation assays

In vitro phosphorylation was carried out with 0.5 μg of purified recombinant substrate protein and 0.5 μg of purified GST-StkP in 30 μl of kinase buffer (50 mM Tris-HCl, 5 mM MgCl_2_, 100 μM ATP, 1mM DTT, pH 7.5). The reaction was started by the addition of ATP and stopped after 60 min of incubation at 37°C by the addition of 5x Laemmli SDS sample buffer. Samples were separated by standard Tris-glycine-SDS polyacrylamide gel electrophoresis (PAGE) gels and electroblotted onto a nitrocellulose membrane. Phosphorylated proteins were detected with an anti-phosphothreonine polyclonal antibody (1∶1,000; Cell Signaling) and a goat anti-rabbit immunoglobulin G secondary antibody conjugated to horseradish peroxidase (1∶2,500; Invitrogen). Detection was performed with an enhanced chemiluminescence substrate (SuperSignal West Pico Chemiluminescent Substrate; Pierce) and Hyperfilm CL film (GE) using exposures of between 1 and 10 min. The pRSET-*divIVA*_spn_ plasmid was generously provided by Dr. Orietta Massidda (Università degli studi di Cagliari, Italy) [78].

### In vivo phosphorylation assays

The RC838 (*comE-his*) and RC839 *(ΔstkP comE-his*) strains (S1 Table) were grown in 2 l of ABM (pH 7.8) at 37°C until OD_600nm_ 0.3. Half of the cultures (1 l) were centrifuged for 10 min at 5,000 x *g*, snap-frozen in liquid-air and stored at -80°C. The remaining 1 l was centrifuged as before, resuspended in ABM (pH 6.0), incubated at 37°C for 10 min, and finally centrifuged for 10 min at 5,000 x *g*, snap-frozen in liquid-air and stored at -80°C. Cell pellets were thawed in ice and resuspended in 10 ml of L8 buffer (100 mM NaH_2_PO_4_/10 mM Tris·HCl, pH 8.0/150 mM NaCl/20 mM imidazole/20mM PMSF/8 M urea) supplemented with MS-SAFE protease/phosphatase inhibitor cocktail (Sigma-Aldrich) and lysed by stirring for 1 h followed by sonication. The lysate was cleared by centrifugation at 15,000 *g* for 20 min, and the supernatant was added to a column packed with to 0.5 ml of Ni-NTA resin (Qiagen) equilibrated in L8 buffer. The column was washed sequentially with 10 ml of LX buffer (L buffer with X = 8, 6, 4, 2, and 0 M urea), and bound ComE-His_6x_ protein was eluted with 2 ml of L0 buffer containing 500 mM imidazole. Protein samples (approximately 0.5 μg ComE-His) were separated by SDS-PAGE, and the gels were stained with ProQ Diamond (Invitrogen) to detect phosphorylated ComE-His, followed by SYPRO Ruby (Invitrogen) total protein staining. Gels were imaged under fluorescence mode in a Typhoon FLA 9500 scanner (GE) and protein bands were quantified using ImageQuant software (GE).

### Identification of phosphorylated residues by nano-LC-MS/MS analysis

Identification of the phosphorylation site was carried out by nano-LC-MS/MS analysis as previously described [79]. Protein bands were in-gel-digested overnight with sequencing grade trypsin (Promega) at 37°C, desalted using micro-reverse phase columns (C18 Omix tips, Varian), vacuum dried and resuspended in 0.1% formic acid (v/v) in water. Tryptic peptides were injected into a nano-HPLC system (Proxeon Easy nLC, Thermo) fitted with a trap column (Easy-column C18 2 cm x 100 um ID). Posteriorly, the samples were separated on a reverse phase nano-column (Easy-Column C18 10 cm x 75 um ID; Thermo) using a linear gradient of acetonitrile 0.1% formic acid (0–45% in 70 min) at a flow rate of 400 nL/min. Mass analysis was performed using a linear ion trap mass spectrometer (LTQ Velos, Thermo) in a data-dependent mode (full scan followed by MS/MS of the top 5 peaks)[79]. Raw data was analyzed using the Proteome Discoverer software package (v.1.3.0.339, Thermo), and Sequest search engine, with the following parameters: enzyme: trypsin; maximum missed cleavage: 2; precursor mass tolerance: 1 Da; fragment mass tolerance: 0.8 Da; Ser/Thr/Tyr phosphorylation and methionine oxidation as dynamic modifications. Searches were performed using a *Streptococcus pneumoniae* database downloaded from UniProt (17/5/2017) and including the His tag-ComE sequence. For phosphosite localization, the phosphoRS algorithm was used and the spectra of phosphorylated peptides were manually inspected to corroborate the phosphosite assignment [80].

### EMSA

The promoter region of *comCDE* (255 bp; *pcomC)* was PCR-amplified using the 5’-Cy5 labeled oligonucleotides NGEP516 and NGEP517 (IDT) and purified using the QIAquick PCR Purification Kit (QIAGEN). DNA-binding assays were performed in a total volume of 10 μl containing 50 mM NaCl, 50 mM Tris/HCl pH 7.5, 5% (v/v) glycerol, 7.5 nM Cy5-labeled PCR fragments, 1 mM MgCl_2_, 0.15 mg Poly(dI-dC) (as the non-specific competitor), and varying concentrations of untreated or StkP-treated ComE proteins. In the latter case, phosphorylation of ComE-His_x6_ by StkP was carried out with an equimolar amount of StkP in kinase buffer (50 mM Bis-Tris propane, 5 mM MgCl_2,_ 1mM DTT, 0.1 μM ATP, pH 7.8 or pH 6.0) for 60 min at 37°C. Protein-DNA binding reactions were incubated at room temperature for 30 min, and “frozen” with an equal volume of 2X Stop Solution [40% (v/v) Triethylene Glycol, 10 mM Tris, pH 7.5]. DNA-protein complexes were resolved by electrophoresis in native Tris-Borate-EDTA polyacrylamide gels [10% (w/v)] containing 30% Triethylene Glycol. Gels were run at 4°C for 120 min at constant voltage (25 V cm^-1^) in a 0.5X TBE buffer and scanned in a Typhoon FLA 9500 biomolecular imager (GE) under fluorescence mode. Free and protein-bound DNA were quantified using ImageQuant (GE). The fraction of DNA bound (FB) at each ComE concentration was fit with a standard binding isotherm using Kaleidagraph (Synergy Software), according to the equation: FB = [ComE]/(*K*_*d*_ +[ComE]), where *K*_*d*_ is the apparent equilibrium dissociation constant and reflects the protein concentration required to shift 50% of the labeled DNA fragment.

### Molecular dynamics simulation

MD simulations were carried out with the NAMD program [81], using the CHARMM27 force field [82]. Spherical boundary conditions and a non-bonded cut-off of 12.0 Å with a switching function of 10.0 Å were used. All systems were submitted to structural minimization in vacuum, and then embed in a water sphere for the MD. The temperature was set to 310 K by a Langevin thermostat. MD simulations were run for 40 ns with an integration step of 2 fs. Analysis of the trajectories was performed using VMD software [83]. Crystal structure images were analyzed using PyMOL [84].

### StkP and ComE interaction by FLISA

StkP and ComE binding interactions were assessed by a sandwich fluorescence-linked immunosorbent assay (FLISA) in black 96-well high binding capacity microplates with clear flat-bottoms (Corning #3601). Each well was filled with 50 μL of 10 μg/ml GST-StkP (500 ng of GST-StkP) dissolved in a 0.1 M coating buffer (0.1 M NaHCO_3_/Na_2_CO_3_, pH 9.4) and incubated overnight at 4°C to allow protein adsorption. Wells were rinsed five times with Tris-buffered saline, 0.05% Tween 20, pH 7.4 (TBS-T) and the reactive sites were blocked with 2% w/v bovine serum albumin dissolved in TBS-T for 2 h at room temperature. Wells were washed three times with TBS-T. Different amounts of ComE-His_x6_ (200–1200 ng) were dissolved in 50 μL of 50 mM Bis-Tris-Propane-HCl, 1 mM MgCl_2_, pH 7.8, or in the same buffer but at pH 6.0, and added to StkP-coated wells and incubated for 1h at 37°C. Wells were washed 5 times with TBS-T and incubated for 1h at RT with 50 μL of a Dylight 650-conjugated anti-6X His antibody (Invitrogen MA1-21315-D650) diluted 100-fold in TBS-T. After 5 washes with TBS-T, plates were read in a Typhoon FLA 9500 scanner (GE) under fluorescence mode. Fluorescence (F) was fit using a Kaleidagraph to a standard binding isotherm with the form F = F_max_ [ng ComE/(*K*_*1/2*_ + ng ComE)], where F_max_ is the maximum fluorescence at binding saturation and reflects the maximum binding capacity (B_max_), and *K*_*1/2*_ is the amount of ComE (ng) required to reach half F_max._ The inverse of *K*_*1/2*_ represents an estimate of ComE affinity for the StkP binding sites.

### Protein expression and purification

The *comE* gene was amplified from R801 genomic DNA with the primer pair FhkE/RhkE and cloned into the BamHI/EcoRI sites of the pRSET-A expression plasmid (Invitrogen), yielding pRSET-ComE. *stkP* and *stkP-KD* (kinase domain, amino acids 1–282) were amplified with primer pairs FstkP-ex/Rstk-ex and FstkP-ex/ Rstk-kd, respectively, and cloned into BamHI/EcoRI sites of pGEX-4T1 expression plasmid (GE) to generate pGEX-StkP and pGEX-StkP-KD. Plasmid pTrc-LytA(N) expressing the amino-terminal region of LytA (1–206) was obtained by cloning a *lytA* fragment generated by PCR amplification with primers F_lytA1_/Rl_ytA2_ into the BamHI/EcoRI sites of pTrcHis2A (Invitrogen). Mutations were introduced in pRSET-ComE by Quickchange site-directed mutagenesis (Agilent), employing primer pairs NGEP514/NGEP515, NGEP75/NGEP76 and NGEP77/NGEP78 to obtain pRSET-ComE(D58E), pRSET-ComE(T128A), and pRSET-ComE(T128E), respectively. In the same way, the K42M mutation was introduced in pGEX-StkP with primers NGEP770/771 to give pGEX-StkP(K42M).

Soluble His_6X_-tagged LytA(N) and ComE-His_x6_ proteins were purified from the *E*. *coli* BL21(DE3) strain co-transformed with either pTrc-LytA(N) or pRSet-ComE derivatives and chaperone expression plasmids pBB540 and pBB550 [2,85]. *E*. *coli* cells were grown on 800 ml of Terrific broth and induced with 100 μM IPTG according to de Marco [85]. His-tagged proteins were purified from protein lysates obtained by sonication using an NTA-Ni^2+^ resin (Qiagen) following the manufacturer’s protocol. Eluted protein was further purified by gel filtration using a HiPrep 16/60 Sephacryl S-200 HR column mounted in a ÄKTA purifier system (GE). ComE containing fractions were pooled, concentrated with an Amicon Ultra-4 centrifugal filter (Millipore), and dialyzed against the storage buffer [50 mM Tris, 200 mM NaCl, 1mM DTT, 50% v/v glycerol, pH 7.5). Samples were snap frozen and stored at -80°C until use. Following exactly the same protocol as above, GST-tagged StkP proteins were purified from the soluble protein fraction of BL21(DE3) cells bearing pGEX-StkP derivatives and plasmids pBB540 and pBB50. In this case, a Glutathione Sepharose resin (GE) was used to retain GST-tagged StkP. DivIVA was purified from BL21 (DE3) cells transformed with pRSET-*divIVA* according to Fadda *et al*. [78]. Purified recombinant *Aequorea victoria* His-tagged GFP protein was purchased from SIGMA.

### Dimeric state of ComE proteins

Native PAGE was used to assess the ComE monomer/dimer ratio. Purified ComE-His_x6_ proteins were diluted in 2x Laemmli sample buffer without 2-mercaptoethanol and SDS and loaded in a 4–20% gradient Bis-Tris precast polyacrylamide gel (GenScript). Electrophoresis was performed at 4°C using Tris-MOPS running buffer without SDS (GenScript) at a constant electric field of 15 V cm^-1^. Proteins were electroblotted onto a PVDF membrane and probed with Dylight 650-conjugated anti-6X His antibody to detect His-tagged ComE. The membranes were imaged under fluorescence mode in a Typhoon FLA 9500 scanner (GE Healthcare), and bands were quantified with ImageQuant software (GE Healthcare).

### RNAseq analysis

Cells were initially grown in THYE medium at pH 7.8 until OD_600nm_ ~0.3 (log phase), centrifuged at 14,000 g for 10 min at 4°C, resuspended in the same volume in ABM at pH 6.0 (Piñas *et al*, 2008) and incubated a 37°C for 1h. Then, cells were centrifuged at 14,000 x *g* for 10 min at 4°C, resuspended in a 1/10 vol of lysis buffer (DOC 1% in 0.9% Na Cl) and incubated 3 min a 37°C until complete lysis. Total RNA was purified by TRIzol reagent according to the manufacturer's instructions (Fisher Scientific) from three biological replicates for *wt* and the *comE*^*T128A*^ mutant. Posteriorly, we used the Ribopure Bacterial RNA Purification Kit (Ambion) following the manufacturer's protocol, with the contaminant DNA being removed using the provided Dnase. rRNA was depleted from 8μg of total RNA using the MICROBExpress Bacterial mRNA Enrichment Kit (Ambion), and then the transcriptome libraries were prepared with TruSeq Stranded RNA Library Preparation Kit (Illumina) following the manufacturer's instructions. Briefly, enriched mRNA was fragmented using reagents provided with the kit, and this was followed by first-strand cDNA synthesis and second-strand generation. The libraries were tagged with unique indexes and amplified for a limited number of PCR cycles followed by quantification and qualification using the DNA High Sensitivity Assay Kit. Samples were sequenced using PE150bp chemistry and the Illumina HiSeq. Reads were trimmed by Trimmomatic 0.36 [86] to generate high-quality reads. Subsequently, these reads of *wt* and the *comE*^*T128A*^ samples were separately aligned to the *Streptococcus pneumoniae* R6 genome using BWA -version 0.7.12-r1039 (bio-bwa.sourceforge.net) at default parameters. The software package SAMtools (http://samtools.sourceforge.net/) was used to convert the sequence alignment/map (SAM) file to a sorted binary alignment/map (BAM) file. The mapped reads ratio (MRR) to the reference in each dataset was calculated by applying the flagstat command of SAMtools software to the BAM file.

### Differential gene expression

The aligned reads were assembled by Cufflinks (version-2.2.1), and then the differentially expressed genes were detected and quantified by Cuffdiff, which is included in the Cufflinks package, using a rigorous sophisticated statistical analysis. The expression of the genes was calculated in terms of FPKM (Fragment per kilobase per million mapped reads). Differential gene expression analysis was carried out between *wt* and the *comE*^*T128A*^ samples.

### qRT-PCR

cDNA was synthesized from 2 μg RNA using the ProtoScript II First Strand cDNA Synthesis Kit (NEB) following the manufacture's protocol. cDNA was cleaned using the QIAquick PCR Purification Kit (Qiagen). Genes were amplified using the oligos listed in the S2 Table and FastStart Essential DNA Green Master Mix (Roche) following the manufacturer's protocol. Expression was determined relative to AU0158 normalized by *gyrA* (spr1099) expression using the ΔΔCt method [87]. The *gyrA* had a similar expression by RNA-Seq for *wt* and the *comE*^*T128A*^ mutant, and this had been used to normalize the expression in *S*. *pneumoniae* in other studies [88].

### Intracellular survival assays

The assays to determine the intracellular survival of pneumococci were performed as reported previously [33], but with modifications. Briefly, 3.0 × 10^5^ of A549 cells per well were seeded in 6 well plates and cultured in DMEM supplemented with 10% of fetal bovine serum (FBS) and incubated for 12 h. Pneumococci were grown in THYE to the mid-log phase (OD_600nm_ 0.3) and resuspended in DMEM (with 10% FBS). Infection of cell monolayers was carried out using a multiplicity of infection (MOI) 20:1. Bacterial internalization after incubation and washes with extracellular antibiotics was approximately 1%, and the occurrence of apoptosis/necrosis caused by pneumococcal infection quantified by flow cytometry (Annexin V/propidium iodide labeling kit; Invitrogen) was approximately 5–10% for all time points analyzed. A549 cells were incubated 3 h with pneumococcal strains and cells were washed three times with phosphate-buffered saline (PBS) and fresh DMEM (without FBS) containing 150 μg/ml potassium penicillin G (Sigma P7794) and 900 μg/ml gentamicin sulfate (US Biological G2030). After a 20 min rest period, cells were washed three times with PBS. The eukaryotic cells were lysed by centrifugation for 5 min at 10,000 rpm and the bacterial pellet was resuspended in THYE medium. The number of internalized bacteria at different time points was quantified after serial dilutions and plating on BHI 5% sheep blood agar plates with incubation for 16 h at 37°C. The time scale referred to the time after elimination of the extracellular bacteria by antibiotic treatment. A 100% survival was defined after 20 min of antibiotic treatment (S1 Fig), and all the samples were referred to this point to calculate the respective percentages.

### Hydrogen peroxide determination

For the detection of H_2_O_2_ released by bacterial cells, the phenol red oxidation microassay was used. Briefly, cells were grown in BHI to the mid-log phase (OD_600nm_ 0.3). Posteriorly, cells were centrifuged at 10,000 x *g* for 5 min, resuspended in Todd Hewitt broth THB (pH 6.0) and incubated by 1 h at 37°C. Aliquots were taken and serially diluted to determine viable cells by plating in BHI-blood agar. Other aliquots were centrifuged at 10,000 x *g* for 5 min, and 100 μl of supernatants were transferred to multiwell plates and mixed with the same volume of PRS buffer (NaCl 140 mM, dextrose 5.5 mM, phenol red 280 μM, and horseradish peroxidase 8.5 U/ml in phosphate-buffered saline, pH7.0). Reactions were incubated for 90 min at 37°C and the reaction was stopped with 10 μl of 1 N NaOH, and the reactive wells were read in a microplate reader (Bio-Rad) with a 595-nm filter. Assays were performed in triplicate and results are expressed as mmoles of H_2_O_2_ released by 10^6^ cells.

### Hydrogen peroxide susceptibility assays

Bacterial strains were grown until OD_600nm_ in BHI and aliquots were treated with H_2_O_2_ 20 mM (final concentration). Every 30 min, aliquots were taken and serially diluted to determine viable cells by plating in BHI-blood agar. The percent survival was calculated by dividing the CFU of cultures after exposure to H_2_O_2_ by the CFU of the control tube without H_2_O_2_. Assays were performed in triplicate and results are shown survival percentage at different time points.

### Limited proteolysis assays

Limited proteolysis with proteinase K was carried out in a 30 μl reaction volume with 3 μg of ComE or ComE^T128E^ and 6 ng of the proteinase K in 10 mM Tris, 1 mM CaCl_2_, pH 7.5, for 30 min at room temperature. Reactions were stopped with 5 mM PMSF, 5 mM EDTA and 1X Laemmli loading buffer, and immediately boiled for 5 min. Digestions with trypsin were performed under the same conditions as before but in 100 mM Tris, pH 8.5. Trypsinized samples were boiled immediately for 5 min after stopping the reactions with 5 mM PMSF and 1X Laemmli loading buffer. The extension of protein digestion was verified by 12% SDS-PAGE followed by SYPRO Ruby staining.

### Accession numbers

The RNA-seq data generated from this study are deposited at the NCBI SRA under the accession numbers SAMN08473835 and SAMN08473836.

## Supporting information

S1 Fig Determination of CSP-induced transformability in *S*. *pneumoniae* mutants. (TIF)

S2 Fig Phenotypical characterization of the *stkP*^*K42R*^ and Δ*mapZ* mutants. (TIF)

S3 Fig StkP and ComE mediate p*comC* activation by acidic stress. (TIF)

S4 Fig (video). Thr^128^ phosphorylation changes the DNA-binding domain of ComE. (MPG)

S5 Fig Conformational changes between ComE and ComE^T128E^ determined by limited proteolysis assays. (TIF)

S6 Fig StkP-mediated phosphorylation of ComE is dependent on pH and alters its DNA-binding affinity. (TIF)

S7 Fig Kinase activity of StkP modifies the ComE phosphorylation and alters EMSA. (TIF)

S8 Fig In vitro interactions between StkP and ComE. (TIF)

S9 Fig Putative DNA binding motif in ComE-regulated genes. (TIF)

S1 Table Plasmids and strains used in this work. (DOCX)

S2 Table Evaluation of acidic-stress induced lysis in *hk* mutants. (DOCX)

S3 Table List of ComE-regulated genes as determined by RNAseq analysis. (XLSX)

S4 Table Primers used in this work. (DOCX)
