# Supplementary figures and images for: Crosstalk between the serine/threonine kinase StkP and the response regulator ComE controls the stress response and intracellular survival of Streptococcus pneumoniae
Source: PLoS Pathog. 2018 Jun 8;14(6):e1007118. doi: 10.1371/journal.ppat.1007118 (PMC6010298; doi:10.1371/journal.ppat.1007118)

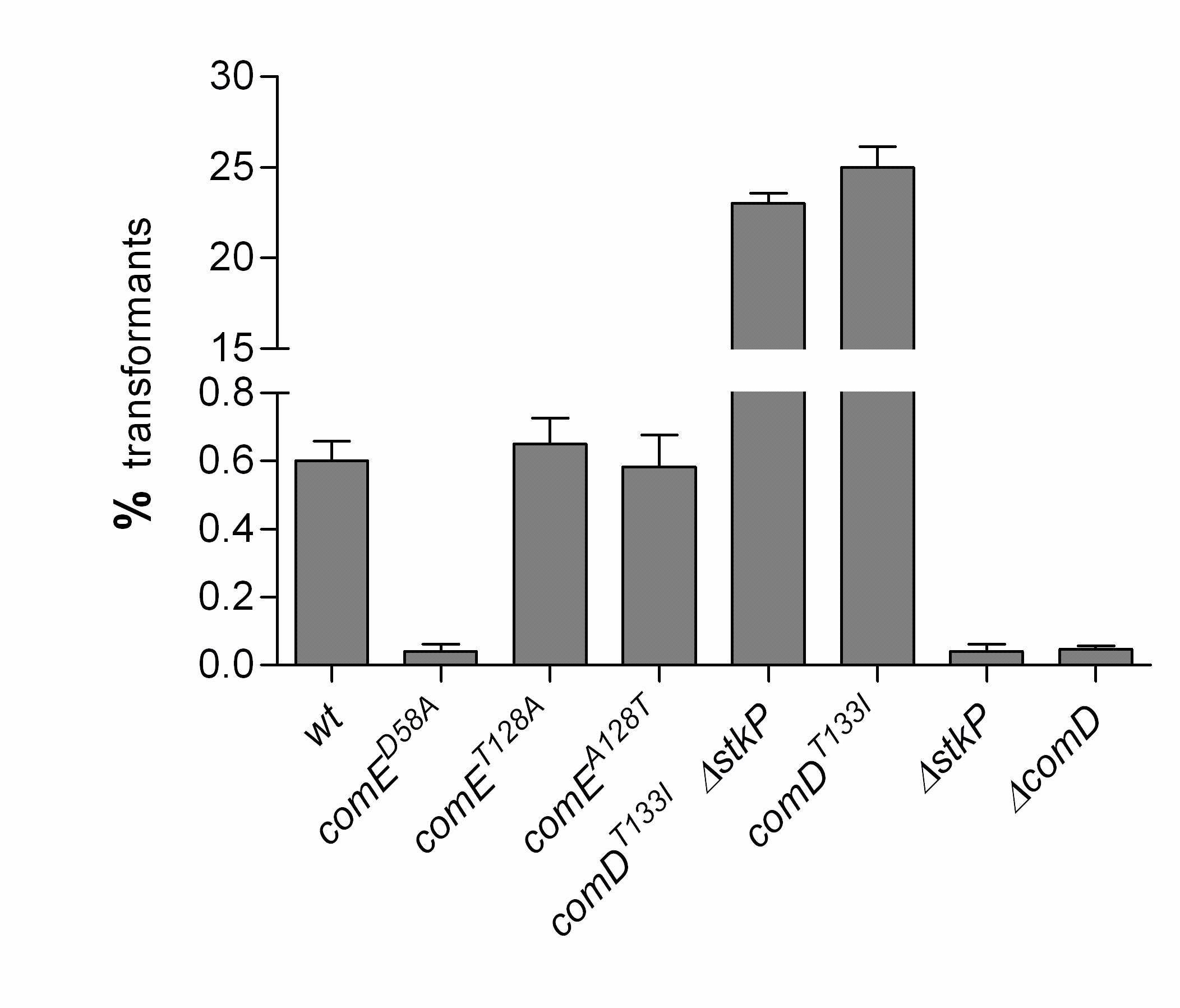

Supplement: S1 Fig — (TIF) [file ppat.1007118.s001.tif]

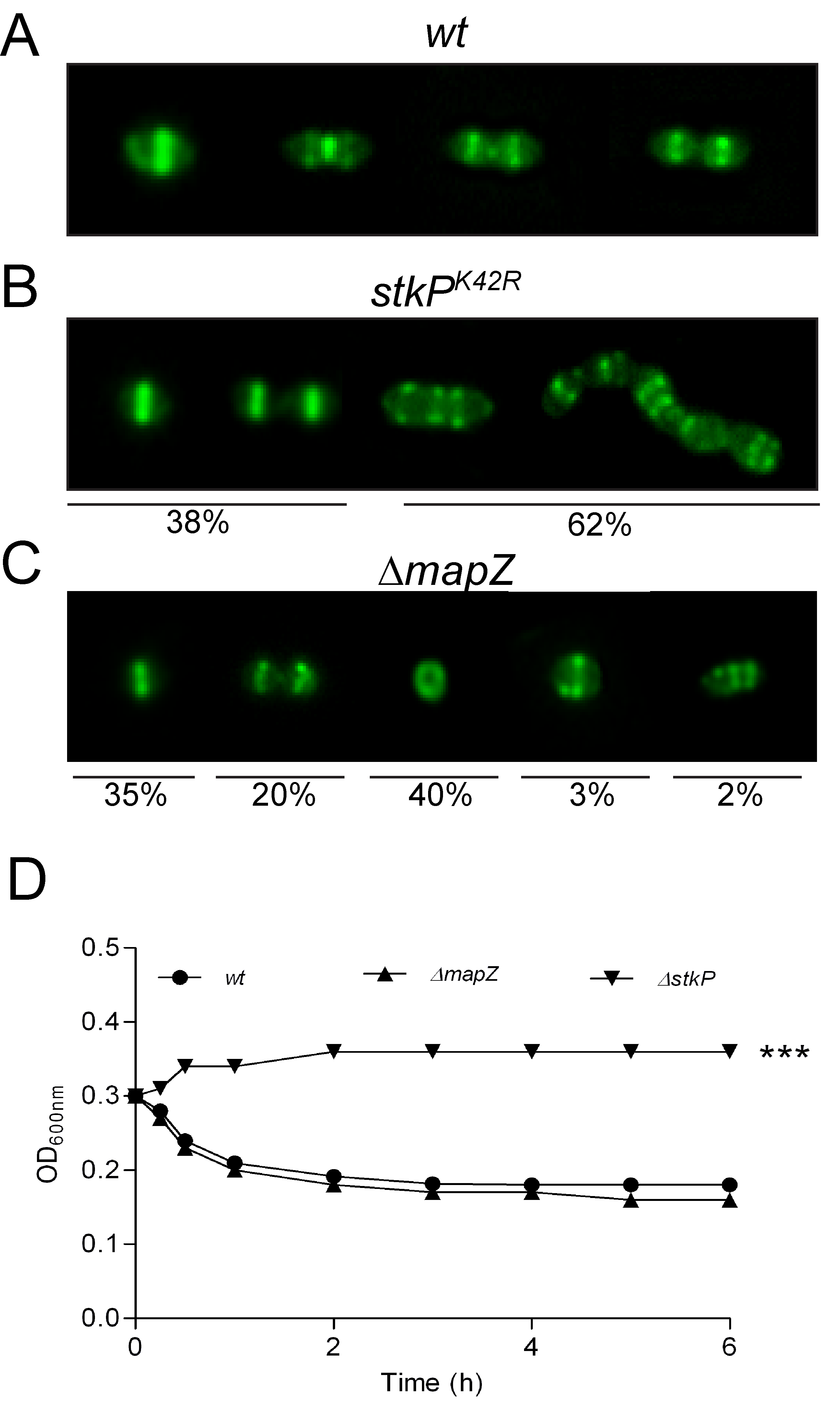

Supplement: S2 Fig — (TIF) [file ppat.1007118.s002.tif]

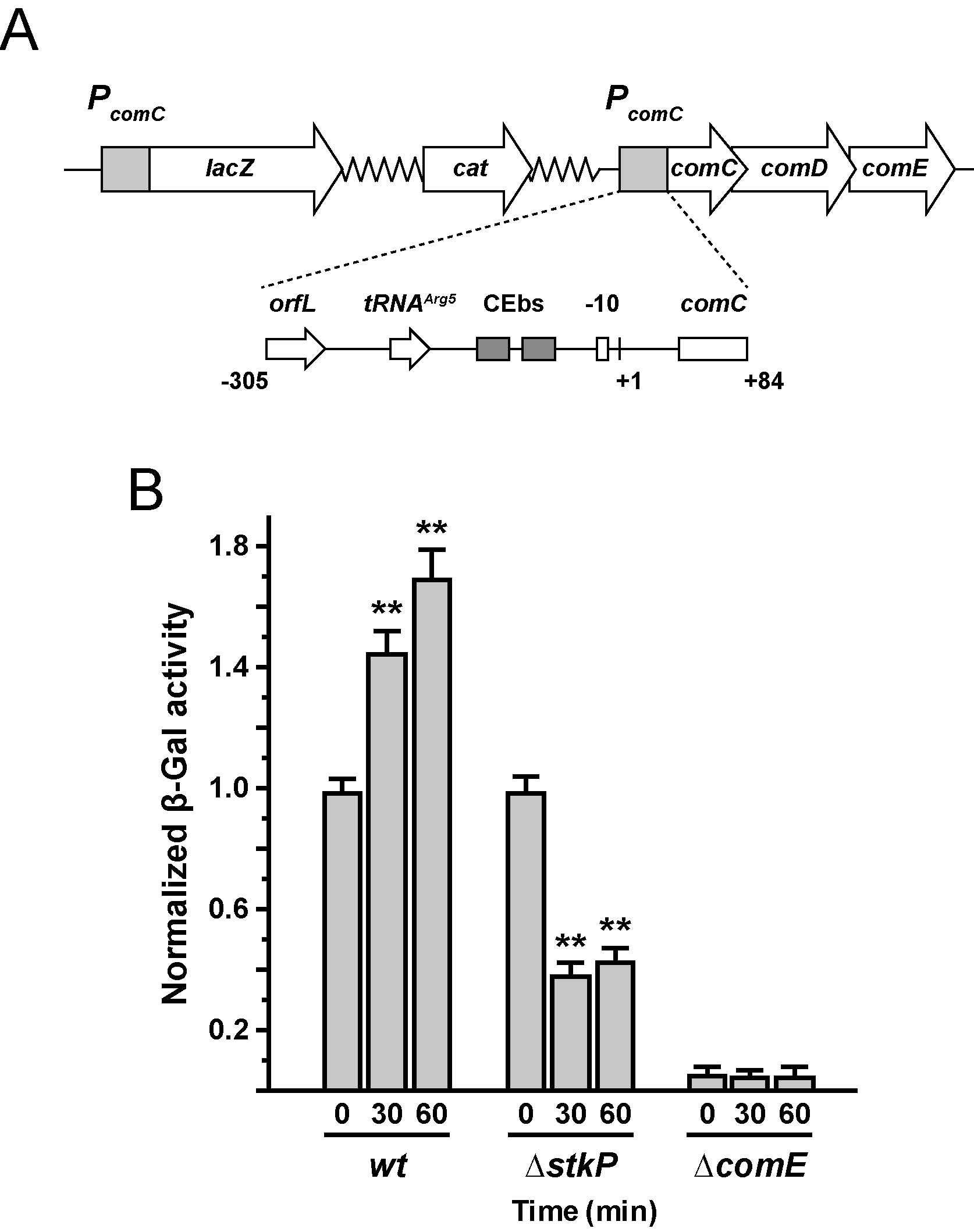

Supplement: S3 Fig — (TIF) [file ppat.1007118.s003.tif]

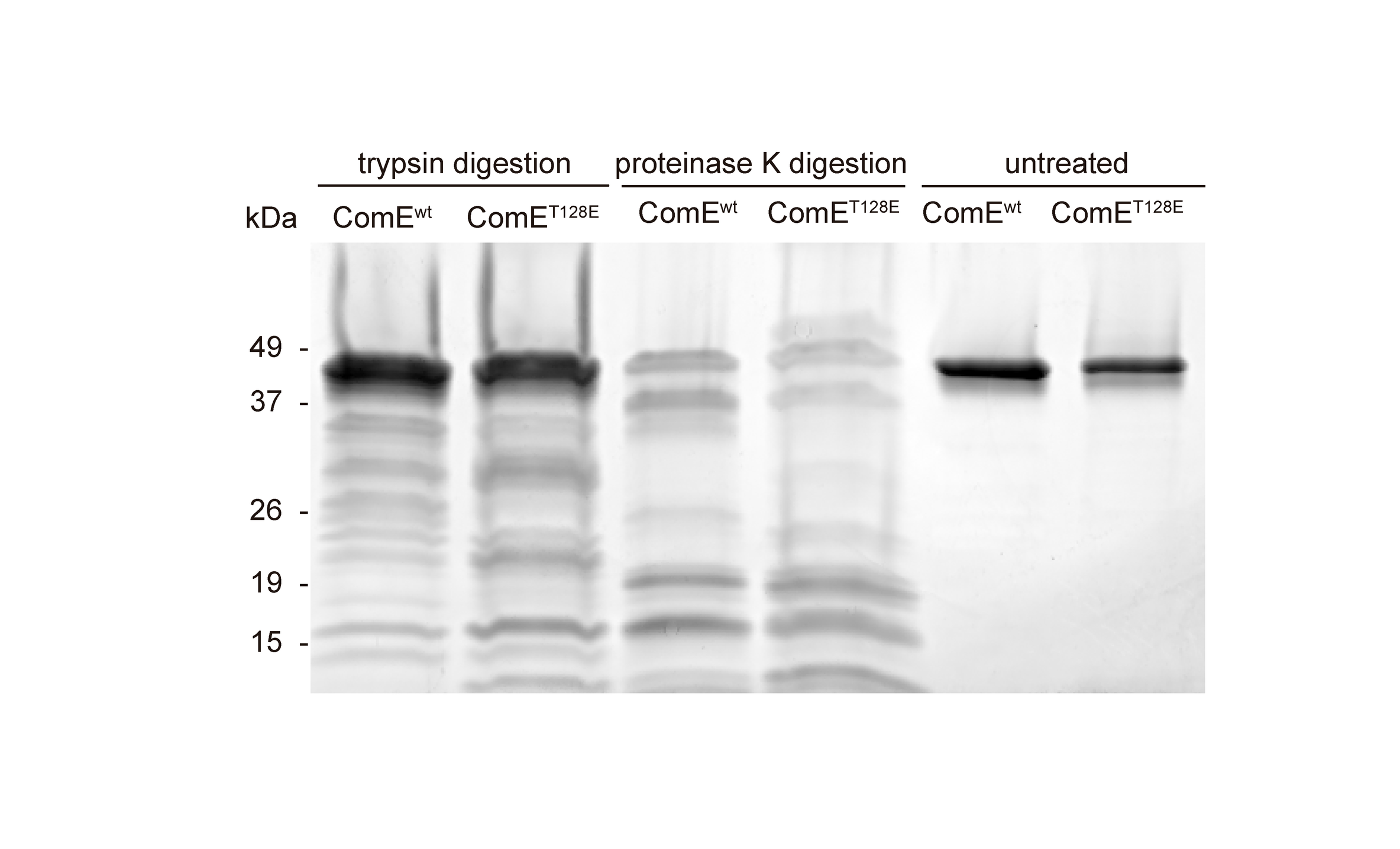

Supplement: S5 Fig — (TIF) [file ppat.1007118.s005.tif]

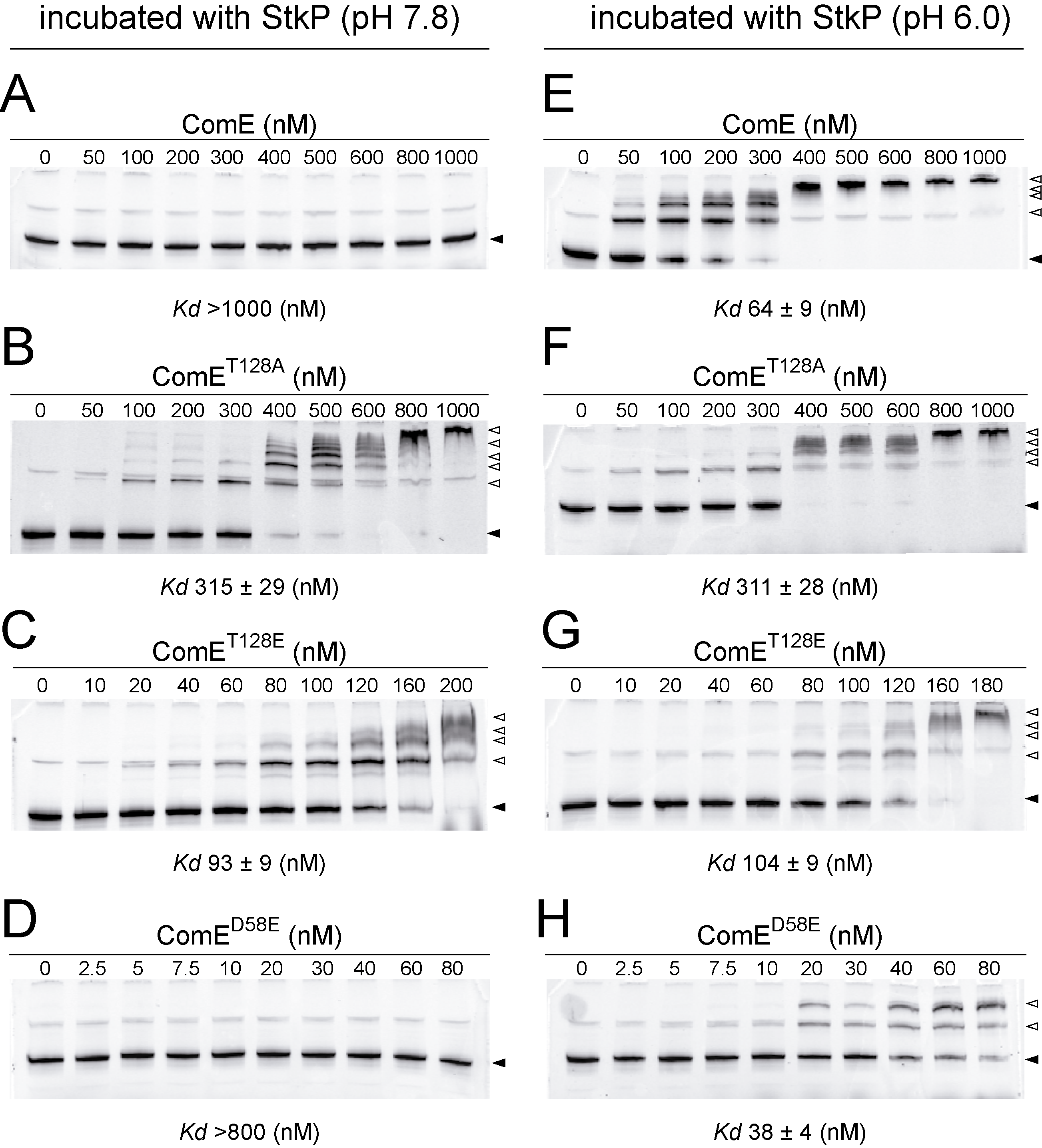

Supplement: S6 Fig — (TIF) [file ppat.1007118.s006.tif]

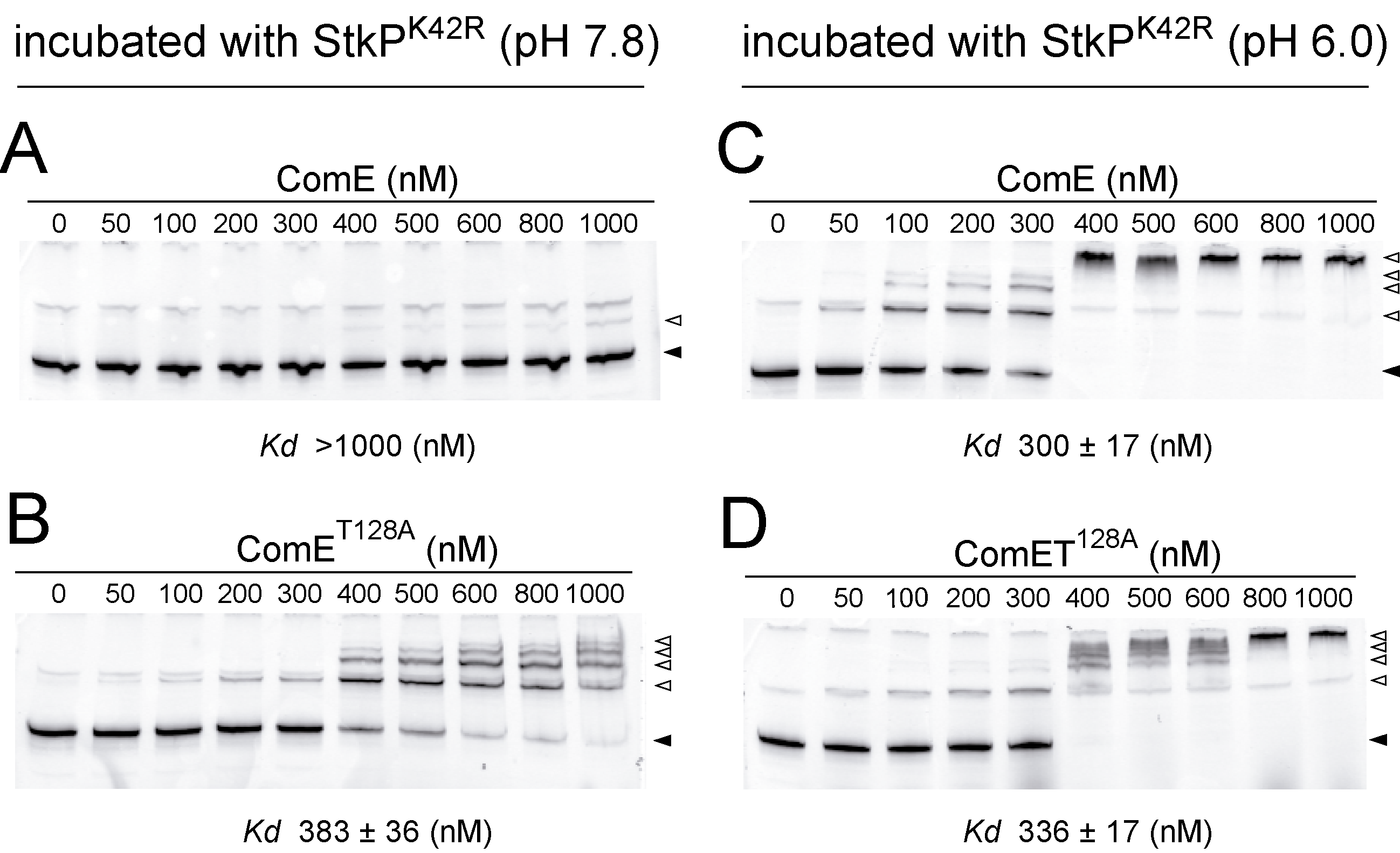

Supplement: S7 Fig — (TIF) [file ppat.1007118.s007.tif]

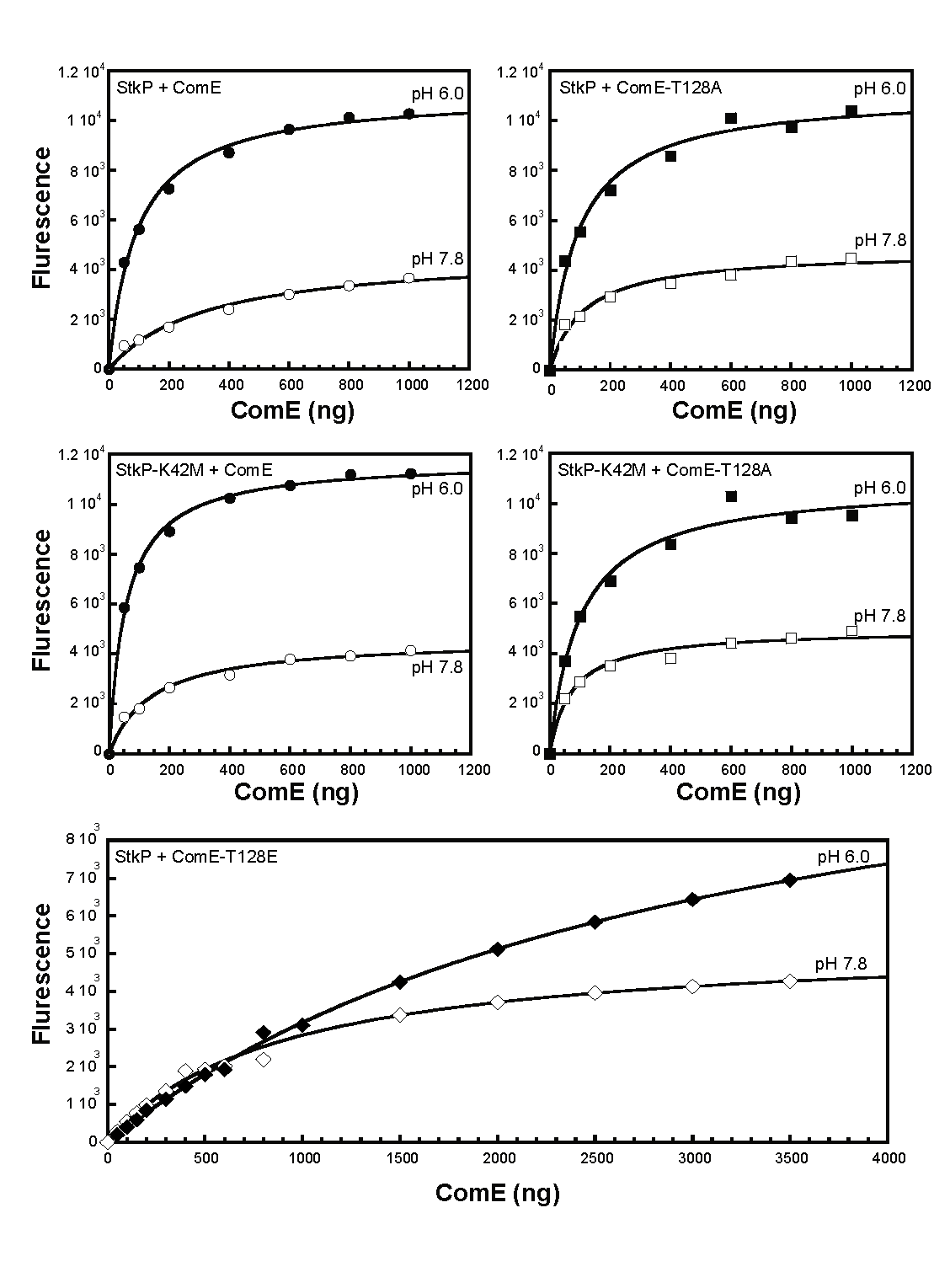

Supplement: S8 Fig — (TIF) [file ppat.1007118.s008.tif]

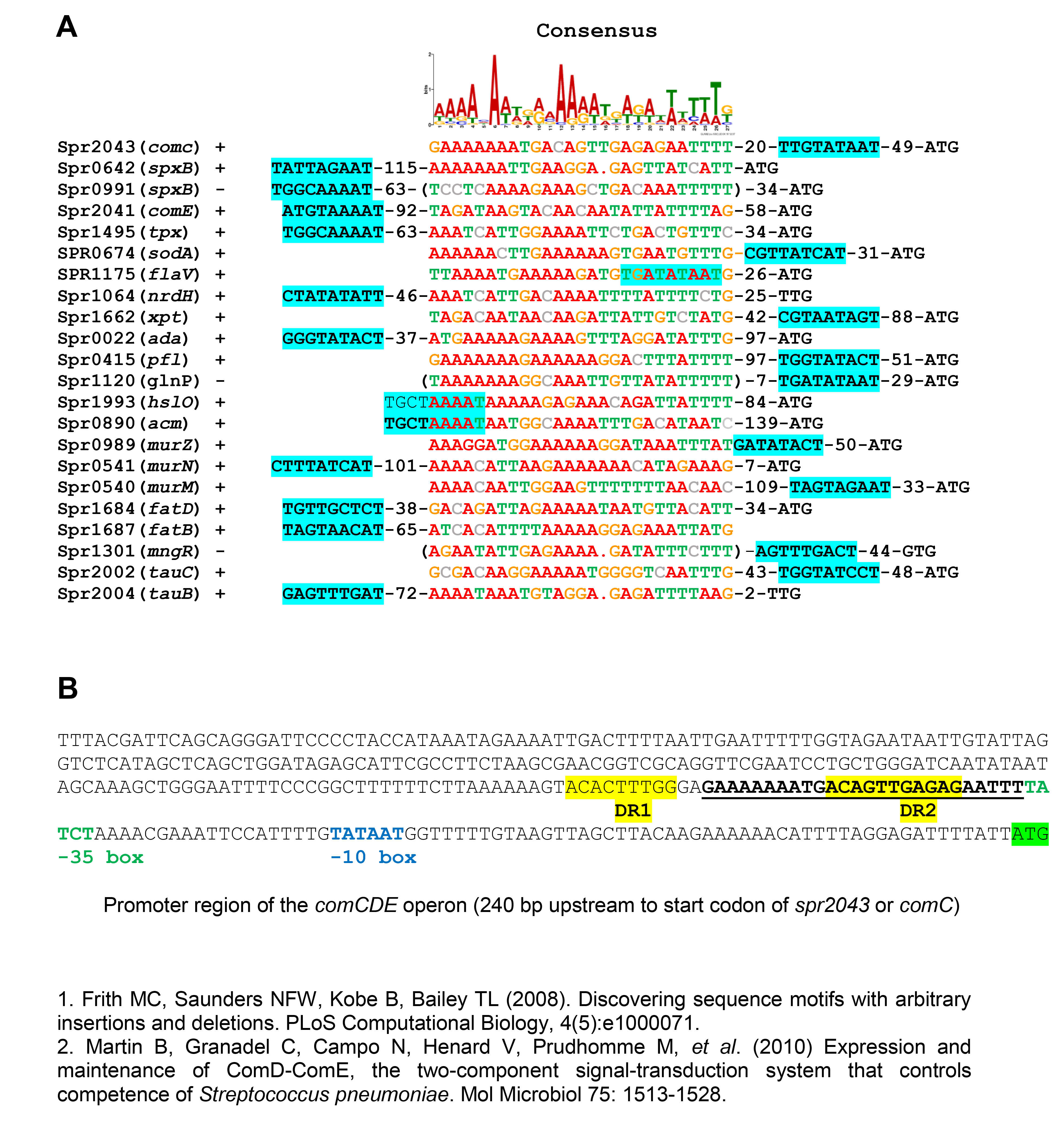

Supplement: S9 Fig — (TIF) [file ppat.1007118.s009.tif]
