# Supplementary material for: Crosstalk between the serine/threonine kinase StkP and the response regulator ComE controls the stress response and intracellular survival of Streptococcus pneumoniae
Source: PLoS Pathog. 2018 Jun 8;14(6):e1007118. doi: 10.1371/journal.ppat.1007118 (PMC6010298; doi:10.1371/journal.ppat.1007118)
Supplement: S1 Table — (DOCX) [file ppat.1007118.s010.docx]

| **Table S1.** Bacterial strains and plasmids used in this work | | |
| --- | --- | --- |
| ***Strains or plasmids*** | ***Relevant characteristics*** | ***References*** |
| **Strains** |  |  |
| *S. pneumoniae* |  |  |
| R801 | R6 derivative; *hexB* Sm^S^ | 1 |
| R806 | R801, but *rpsL1* obtained by transformation of PCR product amplified by FrpsL and RrpsL primers from the CP1296 chromosomal DNA | This work |
| RC820 | R801, but *lytA:: ery*; Ery^R^. | 2 |
| RC821 | R806, but ∆*lytA* obtained by Janus cassette system (amplified from CP1296 chromosomal DNA) and pneumococcal DNA amplified with the FlytA1-Janus, RlytA1-Janus, FlytA2-Janus and RlytA2-Janus primers | This work |
| RC830 | R806, but ∆*comE::km,*  obtained by Janus cassette system (amplified from CP1296 chromosomal DNA) and pneumococcal DNA amplified with the FcomE1, RcomE1, FcomE2 and RcomE2 primers | This work |
| RC831 | R801, but *comE^D58A^* | 3 |
| RC836 | R801, but *comE^T128A^* obtained by transformation of pRSET-comE^T128A^. Mutants were checked by PCR with the FcomE‐HA/RcomET128Ack primers and DNA sequencing. | This work |
| RC837 | RC836, but *comE^A128T^* obtained by transformation of pRSET-comE^A128T^. Mutants were checked by PCR using the FcomE-H6/RcomE-ck-rev primers and DNA sequencing. | This work |
| RC838 | R806, but *comE-His* obtained by transformation with the pEVP3-comE-His plasmid. Transformants were recovered in Cm^R^ agar plates, and construction was checked by PCR and DNA sequencing | This work |
| RC839 | RC838, but ∆*stkP* obtained by Janus cassette system (amplified from CP1296 chromosomal DNA) and pneumococcal DNA amplified with the Fstk1, Rstk1, Fstk2 and Rstk2 primers | This work |
| RC860 | R801, but *comD^T233I^*obtained by transformation of a PCR product obtained with FmutcomD/RcomDaux primers. Mutation was checked by digestion with HindIII of the PCR using FcomDatg/RcomDaux and DNA sequencing if this fragment. | 2 |
| RC861 | R806, but ∆*comD*, obtained by Janus cassette system (amplified from CP1296 chromosomal DNA) and pneumococcal DNA amplified with the FcomD1, RcomD1, FcomD2 and RcomD2 primers | This work |
| RC870 | R801, but *ciaR::spc*; Spc^R^. | 2 |
| RC873 | RC830, but *ciaR::spc* ; Spc^R^. | 2 |
| RC874 | R806, but ∆*ciaR* obtained by Janus cassette system (amplified from CP1296 chromosomal DNA) and pneumococcal DNA amplified with the FciaR1, RciaR1, RciaR2 and RciaR2 primers | This work |
| RC880 | R801, but *ciaH::spc*; Spc^R^. | 3 |
| RC890 | R801, but *comD^F163X^* | 2 |
| RC900 | R801, but *hk01::ery*; Ery^R^. | 3 |
| RC903 | R801, but *comX1::ery* and *comX2::tet*; Ery^R^ Tet^R^ | 2 |
| RC920 | R801, but *hk02:: km*, Km^R^. | 3 |
| RC930 | R801, but *hk03::ery* ; Ery^R^. | 3 |
| RC940 | R801, but *hk04::ery* , Ery^R^. | 3 |
| RC960 | R801, but *hk06::ery*, Ery^R^. | 3 |
| RC970 | R801, but *hk07::ery*; Ery^R^. | 3 |
| RC980 | R801, but *hk08::ery*; Ery^R^. | 3 |
| RC990 | R801, but *hk09::ery* ; Ery^R^. | 3 |
| RC1010 | R801, but *hk10::ery*; Ery^R^. | 3 |
| RC1020 | R801, but *hk11::ery*; Ery^R^. | 3 |
| RC1030 | R801, but *hk13:: ery*; Ery^R^. | 3 |
| RC1040 | R801, but *rr14:: ery*; Ery^R^. | 3 |
| RC1060 | R801, but ∆*stkP* obtained by Janus cassette system (amplified from CP1296 chromosomal DNA) and pneumococcal DNA amplified with the Fstk1, Rstk1, Fstk2 and Rstk2 primers | This work |
| RC1061 | RC874, but ∆*stkP* obtained by Janus cassette system (amplified from CP1296 chromosomal DNA) and pneumococcal DNA amplified with the Fstk1, Rstk1, Fstk2 and Rstk2 primers | This work |
| RC1062 | R801, but *stkP^K42R^* by transformation with pTOPO-stkP^K42R^ | This work |
| RC1063 | RC860 ( *comD^T233I^*), but ∆*stkP* obtained by Janus cassette system using PCR products amplified from CP1296 chromosomal DNA with the Fstk1, Rstk1, Fstk2 and Rstk2 primers |  |
| RC1080 | R806, but ∆*mapZ*, obtained by Janus cassette system (amplified from CP1296 chromosomal DNA) and pneumococcal DNA amplified with the FmapZ1, RmapZ1, FmapZ2 and RmapZ2 primers | This work |
| RCD900 | R890, but *hk01::ery*, by transformation with pJDChk01; Ery^R^. | This work |
| RCD920 | R890, but *hk02:: km*, by transformation with pPT12-Km Km^R^. | This work |
| RCD930 | R890, but *hk03::ery* by transformation with pJDChk03; Ery^R^. | This work |
| RCD940 | R890, but *hk04::ery* , by transformation with pJDChk04; Ery^R^. | This work |
| RCD960 | R890, but *hk06::ery* , by transformation with pJDChk06; Ery^R^. | This work |
| RCD970 | R890, but *hk07::ery* by transformation with pJDChk07; Ery^R^. | This work |
| RCD980 | R890, but *hk08::ery* by transformation with pJDChk08; Ery^R^. | This work |
| RCD990 | R890, but *hk09::ery* by transformation with pJDChk09; Ery^R^. | This work |
| RCD1010 | R890, but *hk10::ery* , by transformation with pJDChk10; Ery^R^. | This work |
| RCD1020 | R890, but *hk11::ery* , by transformation with pJDChk11; Ery^R^. | This work |
| RCD1030 | R890, but *hk13:: ery* , by transformation with pJDChk13; Ery^R^. | This work |
| RCD1040 | R890, but *rr14:: ery* , by transformation with pJDCrr14; Ery^R^. | This work |
| RCB801 | R801, but *bgaA::km;* Km^R^. | 3 |
| RCB802 | RCB801, but *comCp*::*lacZ-pEVP_3_* (Cm^R^) *comC^+^*, by transformation with pEVP3-*comCp-lacZ* |  |
| RCB832 | RCB802, but ∆*comE::km* | This work |
| RCB1060 | RCB802, but ∆*stkP::km* | This work |
|  |  |  |
| CP1296 | Rx derivative; *hex mal rpsL1 cbp3::kan-rpsL^+^* (Janus) Km^R^ Sm^S^ | 4 |
|  | *∆mapZ* | This work |
|  |  |  |
| *Escherichia coli* |  |  |
| TOP10 | Amp^R^, Kan^R^, *lacZα*+ selection .F^-^ *mcr*A 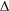(*mrr*-*hsd*RMS-*mcr*BC) 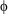80 *lac*Z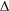M15 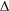*lac*X74 *rec*A1 *ara*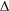139 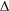(*ara*-*leu*)7697 *gal*U *gal*K *rpsL* (Str^R^) *end*A1 *nup*G | Invitrogen |
| BL21(DE3) | *F- ompT hsdSB (rBmB-) gal dcm (DE3)* | Invitrogen |
|  |  |  |
| **Cloning vectors** |  |  |
| pCR2.1-TOPO | Vector for cloning of PCR products; Ap^R^. | Invitrogen |
| pEVP3 | Integrative vector for *S. pneumoniae;* Cm^R^ | 5 |
| pGEX-4T1 | GST fusion; Ap^R^ | GE |
| pJDC9 | Integrative vector for *S. pneumoniae;* Ery^R^ | 6 |
| pRSET-A | Vector for cloning and protein expression with fusion of His_x6_ tag; Ap^R^. | ThermoFisher |
| pTrcHis2A | Vector for cloning and protein expression with fusion of His_x6_ tag; Ap^R^. | ThermoFisher |
|  |  |  |
| **Plasmids** |  |  |
| pJDChk01 | pJDC9 containing a 0.3 kb *hk01* amplicon | 3 |
| pJDChk03 | pJDC9 containing a 0.42 kb *hk03* amplicon | 3 |
| pJDChk04 | pJDC9 containing a 0.33 kb *hk04* amplicon | 3 |
| pJDChk06 | pJDC9 containing a 0.32 kb *hk06* amplicon | 3 |
| pJDChk07 | pJDC9 containing a 0.26 kb *hk07* amplicon | 3 |
| pJDChk08 | pJDC9 containing a 0.36 kb *hk08* amplicon | 3 |
| pJDChk09 | pJDC9 containing a 0.41 kb *hk09* amplicon | 3 |
| pJDChk10 | pJDC9 containing a 0.36 kb *hk10* amplicon | 3 |
| pJDChk1)1 | pJDC9 containing a 0.39 kb *hk11* amplicon | 3 |
| pJDChk13 | pJDC9 containing a 0.44 kb *hk13* amplicon | 3 |
| pJDCrr14 | pJDC9 containing a 0.3 kb *rr14* amplicon | 3 |
| pBB540 | Contains the *grpE* and *clpB* genes that express *E. coli* chaperones to facilitates recombinant protein expression | 7 |
| pBB550 | Contains the *dnaK, dnaJ and groESL* genes that express *E. coli* chaperones to facilitates recombinant protein expression | 7 |
| pEVP3-*comC-lacZ* | pEVP3 containing a 389 bp *comC* amplicon (promoter region of the *comC* gene) generated with primers FcomCp and RcomCp | This work. |
| pGEX-StkP^K42M^ | contains the stkPK42M allele obtained by site-directed mutagenesis of pGEX-StkP using primers NGEP770 and NGEP771 | This work |
| pRSET-ComE | pRSET-A vector containing the *comE* gene amplified with FcomEex/RcomEex primers from R801 chromosomal DNA Hisx6 | This work |
| pRSET- ComE^D58A^ | contains the *comE^D58A^* amplified with FhkE/RhkE primers from RC831 chromosomal DNA | This work |
| pRSET- ComE^D58E^ | contains the *comE^D58E^* obtained by directed-site mutagenesis using NGEP514/NGEP515 primers and pRSET-ComE as DNA target | This work |
| pRSET- ComE^T128A^ | contains the *comE^T128A^* obtained by directed-site mutagenesis using NGEP75/NGEP76 primers and pRSET-ComE as DNA target | This work |
| pRSET- ComE^A128T^ | contains the *comE^T128A^* obtained by directed-site mutagenesis using NGEP75/NGEP76 primers and pRSET-ComE as DNA target | This work |
| pRSET- ComE^T128E^ | contains the *comE^T128E^* obtained by directed-site mutagenesis using NGEP77/NGEP78 primers and pRSET-ComE as DNA target | This work |
| pRSETA*_divIVA_SPN_* | pRSET-A vector containing the *divIVA* gene, provided by Dr Orietta Massidda (Università degli studi di Cagliari, Italy) | 8 |
| pTrc- N-LytA | pTrcHis2A vector containing the N-terminal region of the *lytA* gene amplified with FlytA1/RlytA1 primers from R801 chromosomal DNA | This work |
| pGEX-StkP | contains the *stkP* gene amplified with FstkP-ex/RstkP-ex primers from R801 chromosomal DNA | This work |
| pGEX-StkP-KD | contains the kinase domain of the *stkP* gene amplified with FstkP-ex/RstkP-KD primers from R801 chromosomal DNA | This work |
| pEVP3-comE-His | contains the *comE*-*His* gene (fused to His tag at C-terminus) amplified with FcomE-Bm/RcomE-His-Sal primers from R801 chromosomal DNA. | This work |
| pEVP3-comET128A-His | contains the comET128A-His gene (fused to His tag at C-terminus) amplified with FcomE-Bm/RcomE-His-Sal primers from RC836 chromosomal DNA. | This work |
| pEVP3-comET128E-His | contains the comET128A-His gene (fused to His tag at C-terminus) amplified with FcomE-Bm/RcomE-His-Sal primers from plasmid pRSET-comET128E. | This work |

Abbreviations: Ap^R^: ampicillin resistance; Cm^R^: chloramphenicol resistance; Ery^R^: erithromycin resistance; Km^R^: kanamycin resistance; Str^R^: streptomycin resistance.

**REFERENCES**

1) **Lefevre JC, Claverys JP, Sicard AM.** Donor deoxyribonucleic acid length and marker effect in pneumococcal transformation. 1979. J. Bacteriol. **138**:80-6.

2) **Piñas GE, Cortes PR, Orio AG, Echenique J.** 2008. Acidic stress induces autolysis by a CSP-independent ComE pathway in *Streptococcus pneumoniae*. Microbiol. (SGM). 154:1300-8.

3) **Cortes PR, Piñas GE, Cian MB, Yandar N, Echenique J.** Stress-triggered signaling affecting survival or suicide of *Streptococcus pneumoniae*. Int J Med Microbiol. 2015 Jan;305(1):157-69.

4) **Sung CK, Li H, Claverys JP, Morrison DA.** An *rpsL* cassette, Janus, for gene replacement through negative selection in *Streptococcus pneumoniae*. Appl Environ Microbiol. 2001 Nov;67(11):5190-6.

5) **Pestova EV, Morrison DA.** Isolation and characterization of three *Streptococcus pneumoniae* transformation-specific loci by use of a *lacZ* reporter insertion vector. J Bacteriol. 1998, 180(10):2701-10.

6) **Chen JD, Morrison DA.** Cloning of *Streptococcus pneumoniae* DNA fragments in Escherichia coli requires vectors protected by strong transcriptional terminators. Gene. 1987. 55(2-3):179-87.

7) **de Marco A, Deuerling E, Mogk A, Tomoyasu T, Bukau B.** Chaperone-based procedure to increase yields of soluble recombinant proteins produced in E. coli. BMC Biotechnol. 2007 . 7():32.

8) **Fadda D, Santona A, D'Ulisse V, Ghelardini P, Ennas MG, Whalen MB, Massidda O.** *Streptococcus pneumoniae* DivIVA: localization and interactions in a MinCD-free context. J Bacteriol. 2007. 189(4):1288-98.
