# Supplementary material for: Crosstalk between the serine/threonine kinase StkP and the response regulator ComE controls the stress response and intracellular survival of Streptococcus pneumoniae
Source: PLoS Pathog. 2018 Jun 8;14(6):e1007118. doi: 10.1371/journal.ppat.1007118 (PMC6010298; doi:10.1371/journal.ppat.1007118)
Supplement: S2 Table — (DOCX) [file ppat.1007118.s011.docx]

**Table S2.** Evaluation of acidic-stress induced lysis in *hk* mutants

| ***hk* mutants** | **ASIL in *wt* background** | **ASIL in *comD^F183X^*  background** |
| --- | --- | --- |
| *hk01::ery* | + | + |
| *hk02::km* | + | + |
| *hk03::ery* | + | + |
| *hk04::ery* | + | + |
| *hk05::spc* | +++ | +++ |
| *hk06::ery* | + | + |
| *hk07::ery* | + | + |
| *hk08::ery* | + | + |
| *hk09::ery* | + | + |
| *hk10::ery* | + | + |
| *hk11::ery* | + | + |
| *hk12** (*comD^F183X^*) | + | NA |
| *∆hk12* (∆*comD*) | + | NA |
| *hk13::ery* | + | + |

(+) lytic profile of R801; (+++) accelerated autolysis compare to *wt*; (NA) not applicable. For a reference of lytic response of the R801 *wt* strain, see Fig. 1.
