# Supplementary material for: Crosstalk between the serine/threonine kinase StkP and the response regulator ComE controls the stress response and intracellular survival of Streptococcus pneumoniae
Source: PLoS Pathog. 2018 Jun 8;14(6):e1007118. doi: 10.1371/journal.ppat.1007118 (PMC6010298; doi:10.1371/journal.ppat.1007118)
Supplement: S4 Table — (DOCX) [file ppat.1007118.s013.docx]

| **Table S4.** Primers used in this work | | |  |
| --- | --- | --- | --- |
| **Primers** | **DNA sequences (5’-3’)** | **Amplified gene** | **Restriction sites** |
| Frpsl | GACGTGCTGACAAATGTTGC | *rpsL* |  |
| RrpsL | AATGTCGACTAGATCTTTCCTTATGCTTTTGGAC | *rpsL* |  |
| FcomE1 | GAGGGATAGAGGACTATTCCTAAGTTT | *comD* |  |
| RcomE1 | GAAGGGGTCGACATCGATTTATCTCTCTAGTCTCACTTGATGTTCA | *comD* | SalI/ClaI |
| FcomE2 | GAAGGGGTCGACATCGATTCTATGAAGACCATCGTTGTCC | *tRNA-glu* | SalI/ClaI |
| RcomE2 | CCCGATCATGACAATAATAAAGG | *tRNA-glu* |  |
| RcomE-His-Sal | TGTTTGTCGACTTAATGATGATGATGATGATGCTTTTGAGATTTTTTCTCTAAAATATCTTT | *comE* | SalI |
| FcomE-Bm | GAATTAGGATCC**ATG**AAAGTTTTAATTTTAGAAGATGTTATTG | *comE* | BamHI |
| FcomD1 | TGCTAAGTTTGAAATGATTGAGTTATCAG | *tRNA-glu* |  |
| RcomD1 | GAATATGTCGACCGGATCCAACCGTCCCAAATCCAAATAAATCC | *comC* | SalI/BamHI |
| FcomD2 | TTAGGGGTCGACGGATCCAGAGATGGAAGGCAGTACATTTAGAC | *comE* | SalI/BamHI |
| RcomD2 | GACCAACGGACCTTCTATCTGTAGC | *comE* |  |
| FmutcomD | GACGAGGACTACCTTTATCCTTTCTTGAAAAAAGTATTTTAAGCTTTA | *comD* | HindIII |
| RcomDaux | GACTGAGCAACCAAACTTCG | *comD* |  |
| FcomDatg | TGGATTTGGGACGGTTATTG | *comD* |  |
| FciaR1 | GAGACTTGTTAGAAGCTGTTCTTGC | *pepN* |  |
| RciaR1 | GATACGGTCGACGGATCCTTATAGGTCATCCTCAACCAATAAGATTT | *pepN* | SalI/BamHI |
| FciaR2 | GAAGGGGTCGACGGATCCTCCGTAAGAAATTAAAGGGAACC | *ciaH* | SalI/BamHI |
| RciaR2 | AAGCAATCATCTCATAGTTTGTGAA | *ciaH* |  |
| Fstk1 | AACTTATAGCACCTGCACTATCG | *phpP* |  |
| RstkP1m | GAAGGGGTCGACGGATCCTTAAACCTTCACTGCCACTTCTTCC | *phpP* | SalI/BamHI |
| Fstk2 | TTAGTCGACGCTACTCCTTAAAAGCAGATGGA | *spr1576* | SalI |
| Rstk2 | ACCGTTGCACATTGAGTACG | *spr1576* |  |
| FcomCp | CCGCTCGAGCTAGTTCTTGTTGAACAAATCTATCG | *comCDE promoter* |  |
| RcomCp | CTAGTCTAGACTTCAAAGCTACAAACTGTTCC | *comCDE promoter* |  |
| FhkE | ACTCGGGATCCATGAAAGTTTTAATTTTAGAAGATGTTATTG | *comE* | *Bam*HI |
| RhkE | CAGCGGAATTCTCACTTTTGAGATTTTTTCTCTAAAATATC | *comE* | *Eco*RI |
| FlytA1 | GGCACGGATCCGATGGAAATTAATGTGAGTAAATTAAG | *lytA* | *Bam*HI |
| RlytA2 | CGCGAATTCTTCCAAGTGCCATTGATTTTCTC | *lytA* | *Eco*RI |
| FlytA1-janus | AAAGTGTGCCAGAACTCTTGCC | *dinF* |  |
| RlytA1-janus | AATATAGTCGACGGATCCCTGTTCTTAATTTACTCACATTAATTTCCAT | *dinF* | *BamHI SalI* |
| FlytA2-Janus | AATATAGTCGACAGGATCCAGGAATGTCTTTCAAATCAGAACAGC | *Spr1753* | *BamHI SalI* |
| RlytA2-Janus | TCCTGAGCTAACTCCACGCA | *Spr1753* |  |
| FstkP-ex | GGCACGGATCCATGATCCAAATCGGCAAGA | *stkP* | *Bam*HI |
| RstkP-ex | ATTATGAATTCTTAAGGAGTAGCTGAAGTTGTTTTAGG | *stkP* | *Eco*RI |
| *Rstk-KD* | ATTATGAATTCTTACTTTCATTTCTACGATTGTAGGA | *stkP* | *Eco*RI |
| NGEP770 | GGGGAAGAAGTGGCAGTGATGGTTCTGAGG | *stkPK42M* |  |
| NGEP771 | CCTCAGAACCATCACTGCCACTTCTTCCCC | *stkPK42M* |  |
| NGEP514 | GAAGTAAATCAGCTTTATTTCCTAGAGATCGATATTCATGGAATTGAGAAAAA | *D58E* |  |
| NGEP515 | TTTTTCTCAATTCCATGAATATCGATCTCTAGGAAATAAAGCTGATTTACTTC | *D58E* |  |
| NGEP75 | AGAATTGAGCAAAATATCTTCTATGCCAAGTCCATGTTACTTGAAAATGAAGATGT | *T128A* |  |
| NGEP76 | ACATCTTCATTTTCAAGTAACATGGACTTGGCATAGAAGATATTTTGCTCAATTCT | *T128A* |  |
| NGEP77 | AGAATTGAGCAAAATATCTTCTATGAAAAATCCATGTTACTTGAAAATGAAGATGT | *T128E* |  |
| NGEP78 | ACATCTTCATTTTCAAGTAACATGGATTTTTCATAGAAGATATTTTGCTCAATTCT | *T128E* |  |
| FcomE-tw | CCGCTCGA*GAATTC*CATGAAAGTTTTAATTTTAGAAGATGTTA | *comE* | XhoI, EcoRI |
| FcomE-H6 | ATGAAAGTTTTAATTTTAGAAGATGTTATTG | *comE* |  |
| RcomE-ckrev | TCATTTTCAAGTAACATACTCTTCGTG | *comE* |  |
| FcomET128A-chk | GAATTGAGCAAAATATCTTCTATGCC | *comE* |  |
| RcomE-ckrev | TCATTTTCAAGTAACATACTCTTCGTG | *comE* |  |
|  |  |  |  |
|  |  |  |  |
|  |  |  |  |
|  |  |  |  |
